# Polymersome magneto-valves for reversible capture and release of nanoparticles

**DOI:** 10.1038/ncomms6010

**Published:** 2014-09-24

**Authors:** P.G. van Rhee, R.S.M. Rikken, L.K.E.A. Abdelmohsen, J.C. Maan, R.J.M. Nolte, J.C.M. van Hest, P.C.M. Christianen, D.A. Wilson

**Affiliations:** 1 High Field Magnet Laboratory, Institute for Molecules and Materials, Radboud University Nijmegen, Toernooiveld 7, 6525 ED Nijmegen, The Netherlands; 2 Institute for Molecules and Materials, Radboud University Nijmegen, Heyendaalseweg 135, 6525 AJ Nijmegen, The Netherlands

## Abstract

Stomatocytes are polymersomes with an infolded bowl-shaped architecture. This internal cavity is connected to the outside environment via a small ‘mouth’ region. Stomatocytes are assembled from diamagnetic amphiphilic block-copolymers with a highly anisotropic magnetic susceptibility, which permits to magnetically align and deform the polymeric self-assemblies. Here we show the reversible opening and closing of the mouth region of stomatocytes in homogeneous magnetic fields. The control over the size of the opening yields magneto-responsive supramolecular valves that are able to reversibly capture and release cargo. Furthermore, the increase in the size of the opening is gradual and starts at fields below 10 T, which opens the possibility of using these structures for delivery and nanoreactor applications.

## Abstract (web summary)

The structure of polymersome vesicles can be controlled during water dialysis, and this can result in the formation of bowl-shaped somatocytes with hollow interiors. Here, the authors show how these somatocytes can be magnetically controlled to capture and release cargo.

## Graphical abstract

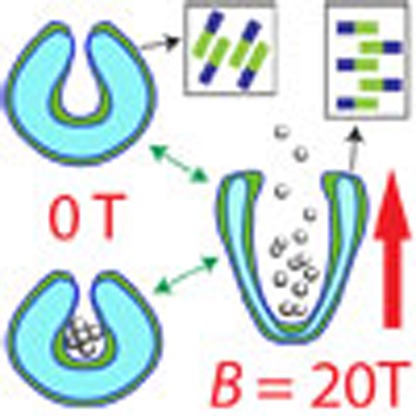

Magnetic manipulation of materials has found many applications, such as in the medical field due to its non-invasive nature^1^,^2^,^3^,^4^,^5^. Some of the most promising applications are the use of magnetic fields as external stimuli in drug delivery (magnetically triggered drug release system)^6^,^7^,^8^,^9^,^10^,^11^, magnetic steering and/or propulsion^12^,^13^,^14^,^15^,^16^,^17^,^18^,^19^,^20^ or in imaging applications through magnetic resonance imaging^16^,^21^,^22^,^23^. Magnetic fields affect all matter and its effect depends not only on the magnetic properties of the material (ferromagnetic, paramagnetic or diamagnetic) but also on the type of the magnetic field applied (homogeneous, inhomogeneous or rotating field)^19^. Furthermore, beside the fact that magnetic fields are non-invasive, their effect is reversible and does not depend on the pH or the ionic strength of the media^2^,^18^. Most of the studies on manipulation of matter in magnetic fields have been focused on ferro- and paramagnetic materials, mainly owing to their relatively strong response in the magnetic field, while diamagnetic materials have been less explored.

Polymersomes, which are bilayer vesicles assembled from amphiphilic block-copolymers have been shown to entrap drugs, proteins, peptides, catalysts or enzymes for a wide range of delivery applications. They also act as biocatalytic nanoreactors and as simple mimics of the eukaryotic cell^24^,^25^,^26^,^27^,^28^,^29^,^30^. The inner nanocavity of the polymersome is not only important for encapsulation but also provides a physical barrier to protect sensitive encapsulated compounds from degradation in analogy to natural biological systems^24^. We have previously shown that the vesicular structure of polymersomes can be further transformed into bowl-shaped polymer stomatocytes by a controlled folding process driven by differences in the osmotic pressure^31^,^32^,^33^,^34^,^35^,^36^. Polymersomes assembled from high glass transition temperature block-copolymers were made flexible and responsive to external stimuli in the presence of an organic solvent. Dialysis of such structures in pure water induced a difference in the osmotic pressure over the membrane and a subsequent change of morphology into stomatocytes. This process continued until the plasticizing solvent was removed and the folded structure was kinetically trapped (Fig. 1). The shape transformation into these hollow bowl-shaped structures generated an extra cavity with high degree of control of the size of the opening, which was used for catalyst entrapment and nanomotor assembly^34^,^35^,^36^. Furthermore, stomatocytes were reversibly plasticized and made flexible when dialysed in a mixture of organic solvent and water^33^,^37^. When exposed for a short time to the organic solvent mixture, the structures retained their morphology with a slight increase in membrane flexibility (Fig. 1), while longer exposure times were shown to change their morphology completely^33^. Addition of water quenched the effect of the organic solvent and vitrified the membrane back into the rigid form (Fig. 1).

Thus, bowl-shaped polymersomes with strict control over the opening were obtained via a kinetically driven process that does not allow a subsequent remote change in the size of the opening under external stimuli^34^. However, bowl-shaped polymersomes capable of reversibly changing the size of their opening under external stimuli and returning to the initial opening size once the stimulus is removed are highly desirable. Such an opening/closing movement effect would allow these supramolecular structures to act in both capture and release mode for their payloads in a reversible and controlled manner, in this way acting as a supramolecular valve system (Fig. 1). The amphiphilic block-copolymers used for the self-assembly of polymersomes are diamagnetic in nature and possess a highly anisotropic magnetic susceptibility *χ* (refs 37, 38). We have recently demonstrated that morphological changes in polymersomes via osmotic stress can be easily detected using magnetic birefringence measurements^37^. Polymersome supramolecular asssemblies are able to respond to a magnetic field via both alignment and possibly also deformation to reversibly generate new conformations and shapes. Although magnetic deformation of vesicles assembled from phospholipids (liposomes)^39^,^40^,^41^,^42^,^43^ and sexitiophene capsules^44^ has been previously demonstrated, no work has been done on the magnetic manipulation of assemblies of polymers and complex bilayer supramolecular structures, such as bowl-shaped polymersomes.

Herein, we report a polymersome magneto-valve system involving the reversible opening and closing of stomatocytes in a magnetic field. The effects of magnetic fields up to 20 T on the morphology of plasticized stomatocytes assembled from poly(ethylene glycol)-polystyrene amphiphilic block-copolymers and the capture of these morphologies at different magnetic strengths are presented. As will be shown below, the size of the opening can be strictly controlled by the strength of the magnetic field *B*, (*B*=|**B**|, where **B** is the field vector) with a concomitant deformation of the bowl-shaped polymersomes from an overall spherical stomatocyte morphology into a prolate shape morphology. Furthermore, the transformation is completely reversible and allows for both controlled capture and release of particles in the magnetic field (Fig. 1).

## Results

### Magnetic deformation

**Figure 2:**
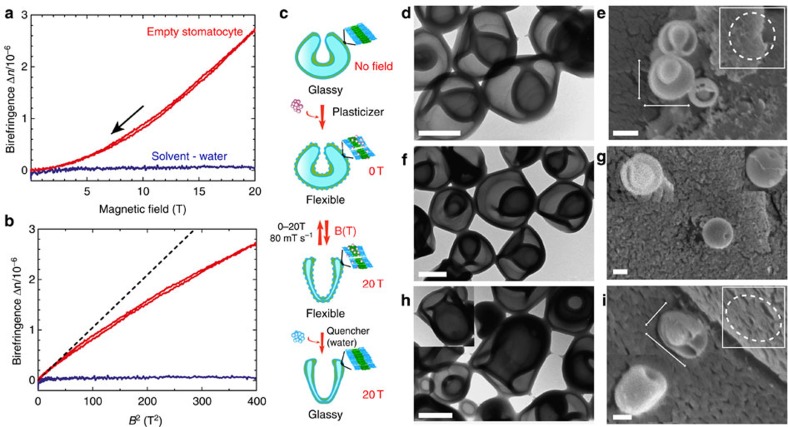
On-line and off-line measurements on the magnetic valve. (**a**) I*n situ* linear birefringence measurements on flexible bowl-shaped stomatocytes in a magnetic field, recorded during both up- and down-sweeps of the field with 80 mT s^−1^ from 0 to 20 T (red line) and on the water solvent mixture (blue line). (**b**) Birefringence as a function of the square of the field strength *B*^*2*^ (T^2^). (**c**) Schematic representation of the deformation in a high magnetic field of spherical narrow-opening stomatocytes into prolate wide-opening structures and their fixation via membrane quenching. (**d**) TEM and (**e**) cryo-SEM of polymer stomatocytes before induced flexibility by reverse dialysis and in the absence of magnetic field. (**f**) TEM and (**g**) cryo-SEM of stomatocytes after 20 min reverse dialysis and magnetic field exposure from 0 to 20 T (80 mT s^−1^) and back to 0 T and vitrified at 0 T for visualization. (**h**) TEM and (**i**) cryo-SEM of polymer stomatocytes obtained after applying the magnetic field cycle from 0 to 20 T (80 mT s^−1^) and vitrification of the membrane at 20 T by water addition for microscopy visualization of the structures. The scale bar for all the TEM and cryo-SEM images represents 200 nm.

Glassy stomatocytes were assembled from a poly(ethylene glycol)_44_-*b*-polystyrene_177_ block-copolymer via a previously reported procedure^35^. Their structure was verified by both transmission electron microscopy (TEM), scanning electron microscopy (SEM) and cryo-SEM techniques, demonstrating their overall spherical bowl-shaped morphology (Fig. 2d,e). Addition of a plasticizing organic solvent (tetrahydrofuran (THF)–dioxane) made the block-copolymer membrane susceptible to manipulation by magnetic fields. This was achieved by reversely dialysing the stomatocyte solution for a short period of 20 min in a mixture of 1:1 (v/v) organic solvent: water, which made the membrane of the polymeric stomatocytes flexible without affecting the shape and the size of the structures (Fig. 2f,g; Supplementary Fig. 1). Two instrumentation techniques were used to observe the effect of the magnetic field on the shape of flexible polymeric stomatocytes. The first method involved the use of magnetic linear birefringence (LB), which provides a direct tool to probe the deformation and alignment of self-assembled structures *in situ* as a function of the magnetic field strength *B*, and was previously shown to be very sensitive to morphological changes of polymersomes that underwent an anisotropic change in shape^37^ (Supplementary Fig. 2). *In situ* birefringence measurements of flexible stomatocytes showed an increase in the birefringence with the magnetic field strength (Fig. 2a,b; Supplementary Fig. 3). The increase of the birefringence with the field was fully reversible and independent of the sweep rate (40 or 80 mT s^−1^). As expected, control experiments on the solvent mixture used for the reverse dialysis did not show any increase or change in the birefringence upon increasing the strength of the magnetic field. The second methodology used to probe the response of the stomatocytes in the magnetic field was electron microscopy (Supplementary Methods). For this *ex situ* technique to be valid, the morphology at different magnetic fields was preserved by quenching and vitrifying the structures in the magnet via water addition. In this way we could investigate the possible deformation effects of the magnetic forces onto the stomatocyte morphology and the size of the stomatocyte opening (Fig. 2c–i). Both (cryo-) SEM and TEM allowed for the visualization of the stomatocytes at magnetic field strengths of 0 and 20 T (Fig. 2f,g; Supplementary Figs 4–7; Fig. 2h,i; Supplementary Figs 8–11). When flexible stomatocytes were placed in the magnet and exposed to a magnetic field from 0 to 20 T and vitrified at 20 T, both TEM and cryo-SEM images clearly showed the deformation of the flexible stomatocytes from a spherical overall shape into a prolate shape morphology, all induced by the magnetic field (Fig. 2h,i; Supplementary Figs 8–11).

Furthermore, an average increase in the size of the opening of the stomatocyte was observed from closed at 0 T to ~39±12 nm at 10 T (measured on 19 stomatocytes) and further increase to 147±20 nm at 20 T (measured on 17 stomatocytes, Fig. 2h,i). However, once the field was removed, TEM and cryo-TEM images showed that the stomatocytes recovered their original overall spherical shape and the narrow size of the opening (compare Fig. 2d,e with Fig. 2f,g; Supplementary Fig. 1 with Supplementary Fig. 5), demonstrating that the magnetic-induced transformation is reversible. TEM and cryo-SEM images of the structures obtained at 0 T after the magnetic sweep showed no change in their overall spherical shape, diameter and size of the opening once the magnetic field was removed (Fig. 2f,g; Supplementary Figs 6 and 7). Furthermore, these electron microscopy images are in full agreement with the magnetic birefringence signals in Fig. 2a,b, which are identical for both the up- and down-sweeps of the magnetic field, demonstrating the reversible deformation of spherical stomatocytes (no birefringence) into prolate stomatocytes with open mouths (large birefringence). Once the field was brought back to 0 T, the down-curve birefringence followed the same trend as the up-curve, decreasing to zero once the field was removed (Fig. 2a,b; Supplementary Fig. 3).

### Mechanism of the magnetic valve

**Figure 3:**
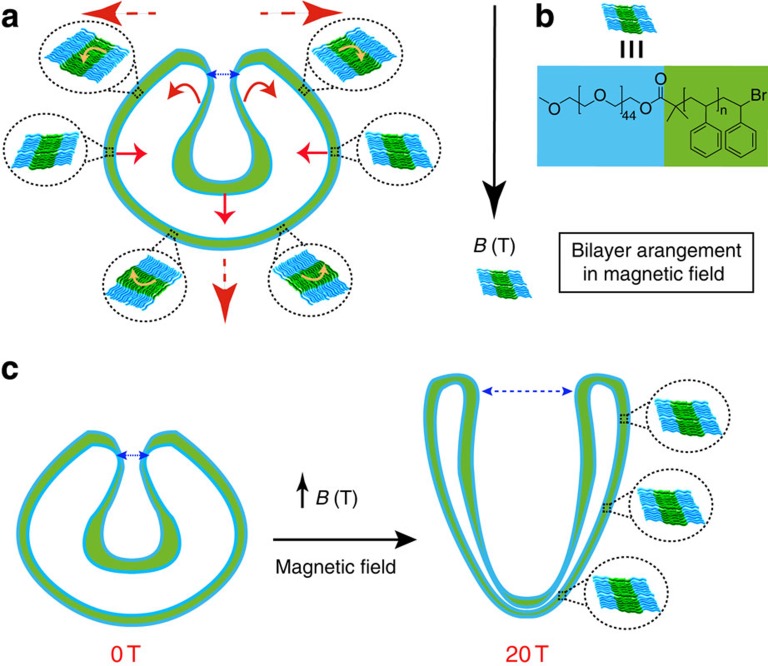
Mechanism of operation of the stomatocyte magneto-valve. (**a**) Representation of the mechanism of magnetic deformation of the stomatocytes and the magnetic forces applied onto the membrane (red arrows). Yellow arrows show the net orientation of the individual polymer chains inside the bilayer to reach the preferred perpendicular orientation to the magnetic field. (**b**) Preferential perpendicular orientation of the bilayer in a magnetic field ***B*** and the chemical structure of the amphiphiles assembled into the bilayer. (**c**) High magnetic field deformation of the stomatocytes from spherical at 0 T into the prolate morphology at 20 T and the resulting increase in the size of the stomatocyte opening at high field (magneto-valve) as a result of the magnetic forces on the membrane.

Both magnetic birefringence measurements and electron microscopy techhniques demonstrated the deformation of the structures from a closed-opening spherical stomatocyte into a wide-opening ellipsoidal stucture. To understand the mechanism of deformation in the presence of a magnetic field, we analysed the anisotropy of the magnetic susceptibility of the poly(ethylene glycol)-polystyrene amphiphile used for the formation of the supramolecular structures by calculating the contributions of each molecular unit within the amphiphile, taking into account its orientation in *x*,*y*,*z* coordinates. The theoretical calculations showed that both the phenyl groups of the PS and the PS backbone contribute to a negative magnetic susceptibility, which means the amphiphile will align perpendicular to the applied magnetic field (see Supplementary Note 1; Supplementary Figs 12–14). The formation of the prolate shape stomatocyte morphology and the magnetic field-induced increase in the size of the opening is therefore due to the preferential perpendicular orientation of the polystyrene blocks in the magnetic field (see Supplementary Note 1) and the resulting magnetic forces applied to the self-assembled membrane (Fig. 3). In a homogeneous magnetic field *B*, the magnetic energy *E* of a polymer with an anisotropic magnetic susceptibility Δ*χ* is given by:




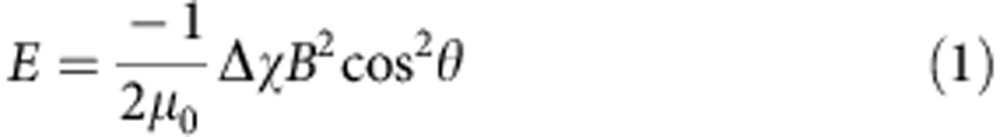




with *μ*_0_ the magnetic permeability of vacuum, *θ* the angle between the polymer backbone and the applied magnetic field and Δ*χ* the difference between the magnetic susceptibility parallel and perpendicular to the polymer backbone (Δ*χ*=*χ*_*//*_–*χ*_⊥_). Since the polymers have a negative magnetic anisotropy, Δ*χ*, their magnetic energy is lowest when oriented perpendicular to the magnetic field. Initially, the magnetic field will align the stomatocyte, since the structure as a whole has an anisotropic diamagnetic susceptibility, which is determined by the distributions of all polymer orientations within the stomatocyte membrane. For instance, a sphere with an isotropic orientational distribution of polymers has no preferential axis of alignment. However, when the distribution of molecular orientations is anisotropic, as it is in a stomatocyte, magnetic alignment can occur. For a stomatocyte, the distribution of polymer orientations is slightly biased towards a perpendicular orientation with respect to the stomatocyte opening. Therefore, the stomatocyte will align in a magnetic field as shown in Fig. 3a,b^37^. After alignment of the stomatocyte, the alignment of the polymers within the membrane can decrease the magnetic energy even further. Although the changes in magnetic energy for one single polymer are neglectable compared with *kT*, a small patch of membrane consisting of many identically oriented polymers will certainly be able to compete with the thermal energy (see Supplementary Note 1), leading to their collective alignment in a magnetic field. The alignment of the polymers in the membrane leads to a deformation of the stomatocyte as shown in Fig. 3c,d and the obvervation of a large magnetic birefringence signal (Fig. 2a,b; Supplementary Fig. 3)^37^. While in the equatorial position the stomatocyte bilayer is already in the preferred orientation, at the rim of the stomatocyte opening, the magnetic field will force the amphiphiles to reorient perpendicular to the field and consequently deform and elongate the structure. At the same time, this results in an increase in the size of the opening with the increase of the magnetic field. Since an increase in deformation will also lead to an increase in bending energy, which counteracts the deformation, the extent of deformation is expected to increase at higher magnetic fields.

### Remote capture and release with the magnetic valve

**Figure 4:**
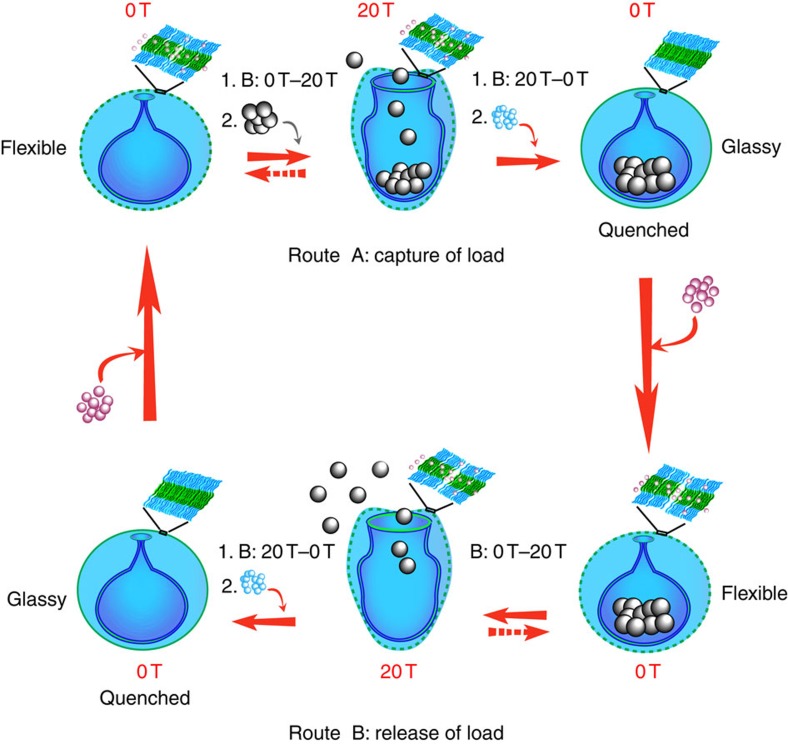
Strategy for capture and release of cargo with stomatocyte magneto-valve. Schematic representation of the reversible and controlled capture and release of particles via deformation of polymeric stomatocytes from an overall spherical stomatocyte shape with narrow opening into a prolate stomatocyte shape morphology with wide opening, all induced by the magnetic field *B* at 20 T (routes A and B). The mechanism of capture and release of the particles is purely diffusive and is based on the diffusion of the particles in or out of the structures during the shape transformation under magnetic field. The pink spheres represent the organic solvent molecules required to create a fluidic membrane that is responsive in magnetic field. The blue spheres represent the water molecules used to vitrify the final morphology into quenched structures and the grey spheres represent platinum nanoparticles used for encapsulation.

The remote reversible change of the stomatocyte opening in a magnetic field gives rise to a magneto-valve nanosystem and the potential to use this dynamic stimulus-responsive behaviour for entrapment and delivery applications in a reversible and controlled manner (Fig. 4). This means that nanoparticles can be entrapped via diffusion during the transition from the large opening stomatocytes at 20 T to closed structures at 0 T (route A, closing the valve) or released in the same way via a diffusive mechanism during the opening of the stomatocyte valve at 20 T (Fig. 4).

**Figure 5:**
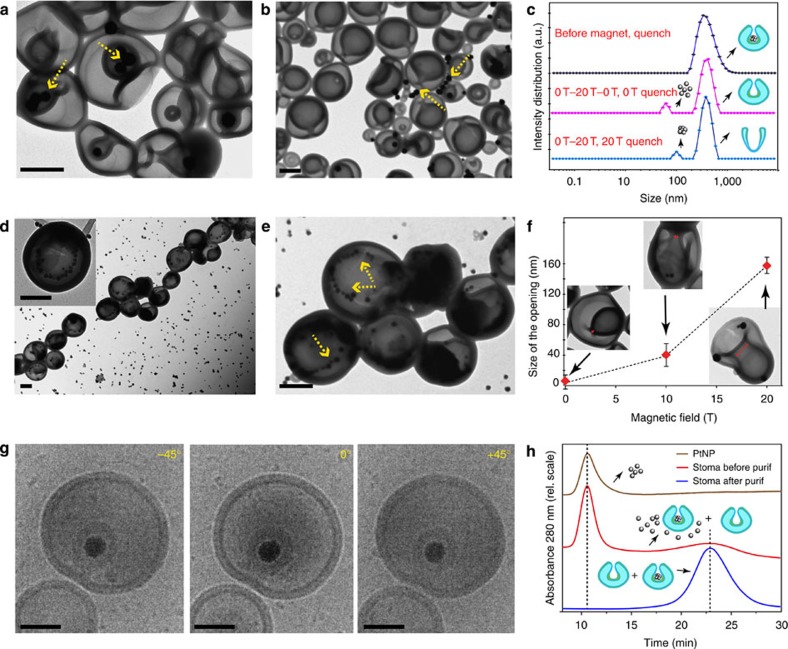
Capture and release of nanoparticles. TEM images of stomatocytes filled with platinum nanoparticles (**a**) before and (**b**) after exposure to a magnetic field of 20 T and back to 0 T (40 mT s^−1^); note the release of the platinum nanoparticles in the medium (marked with yellow arrows). (**c**) Dynamic light-scattering measurements of stomatocytes entrapping platinum nanoparticles before and after deformation in a magnetic field; the release of platinum nanoparticles during the deformation into the wide-opening prolate morphology is shown. (**d**,**e**) TEM images of the stomatocytes entrapping 40-nm platinum nanoparticles at different magnifications. The scale bar for the TEM images is 200 nm. (**f**) Size of the opening of the stomatocytes at 0, 10 and 20 T and their TEM images demonstrating the gradual opening and development of shape asymmetry of the objects during the process. (**g**) Cryo-TEM images of stomatocytes entrapping 60-nm platinum nanoparticles at different angles demonstrating the presence of the particles inside of the structures. The scale bar for the cryo-TEM images is 100 nm. (**h**) Asymmetric field flow fractionation chromatogram of the 60-nm platinum nanoparticles used in the capture experiment, stomatocytes after capture of particles in the magnetic field and after their purification via spin filtering to remove the free platinum nanoparticles. Note the chromatogram after spin filtering shows complete removal of free PtNP. The samples were then used for quantification of the amount of platinum inside of the structures via ICP-MS showing the presence of 2.43±0.11 mg of Platinum per 10 mg polymer (1 ml).

To demonstrate the ability of these structures to reversibly entrap and release loads, platinum nanoparticles (PtNPs) of different sizes (40, 60 and 80 nm in diameter) were synthesized according to our previously reported procedure^35^,^36^,^45^. These particles were added to the open stomatocytes at high field and entrapped during the reversible closing of the valve at zero field (Fig. 4, route A). In addition, nanoparticle-filled stomatocytes were allowed to release their content at high field by opening the magnetic valve in the field at 20 T and keeping the field stable at 20 T for 1 min (Fig. 4, route B). Furthermore, by quenching their morphology inside the magnet at different magnetic field strengths, we were able to detect both open and closed states of the magneto-valve, and observe the entrapment and release processes. When exposed to the magnetic field, the loaded bowl-shaped morphology exhibited a similar reversible increase of the birefringence with respect to the magnetic field, showing no influence of the loaded nanoparticles (Supplementary Fig. 3). TEM analysis of the vitrified samples at 0 T, before and after applying the magnetic field, showed a complete recovery of the overall spherical morphology, and a release of the PtNPs at 20 T (Fig. 5a,b). This observation was further supported by dynamic light-scatterring (DLS) measurements on the loaded stomatocytes before and after applying the magnetic field. The DLS curve of the loaded stomatocytes showed as expected only one distribution around 450 nm. However, this DLS peak appears broad, which suggests a non-uniform distribution of the particles. This is due to the fact that PtNP-loaded stomatocyte samples used for the release experiment in the magnetic field are not 100% filled structures and only 50% of the stomatocytes are filled with nanoparticles. Therefore the sample contains both filled nanoparticles and unfilled while the nanoparticle distribution within the filled stomatocytes is not uniform, some being filled with more particles than others (Fig. 5a; Supplementary Fig. 15). Once the structures were exposed to the magnetic field, a change of their shape from an overall closed spherical morphology to an open prolate shape was expected. As a consequence of the increase of the stomatocyte opening, the entrapped particles were released and an extra peak appeared in the DLS while the stomatocyte structures recovered their uniform shape distribution (Fig. 5c; Supplementary Fig. 16). This is in agreement with the TEM observation of particle release from the stomatocyte cavity (Fig. 5a,b, yellow arrows). When the structures were quenched in the magnet at 20 T, a slight increase in the size of the released PtNPs was observed by DLS, possibly due to some clustering induced by the magnetic field (Fig. 5c; Supplementary Fig. 16). Furthermore, the size of the opening of the loaded stomatocytes was found to gradually increase with increasing the intensity of the magnetic field (Fig. 5f). This shows that the stomatocyte magneto-valve can generate different-sized openings depending on the strength of the magnetic field, while the captured particle/load can be released at different intensities of the field depending on its size.

In a similar manner, we envisaged that nanoparticles could be entrapped during the transition from the open form at high magnetic field (Fig. 4, route A) to the closed form at zero field. Flexible empty stomatocytes were exposed to an increasing magnetic field from 0 to 20 T to generate the wide-open valve morphology while nanoparticles of different sizes were added to the mixture inside the magnet. The samples were kept in the magnet at 20 T for 60 s after which the field was swept back to 0 T and followed by sample quenching at zero field. TEM images of the quenched structures at zero field showed the capture of PtNPs during the transition from the open prolate shape at 20 T to the closed spherical form at 0 T (Fig. 5d,e). The TEM analysis of the images indicated the presence of the nanoparticles inside of the structures and their localization close to the inner membrane of the stomatocyte stomach (yellow arrows, Fig. 5e). All of the stomatocytes in the TEM showed the presence of 40-nm PtNP entrapped inside their nanocavities, however, with a different loading efficiency (Fig. 5d,e), while as expected the encapsulation efficiency was lower for larger particles (60 or 80 nm) (Fig. 5g; Supplementary Figs 20–23). Additional experiments were performed to analyse more quantitatively the amount of platinum entrapped inside of the structures using a combination of techniques such as assymetric field flow fractionation coupled to quasi-elastic light scattering and inductively coupled plasma-mass spectrometry (ICP-MS) (Supplementary Methods; Supplementary Table 1). The field flow fractionation coupled to quasi-elastic light scattering technique allowed for a highly efficient fractionation of the stomatocyte sample obtained during the capture experiment, and simultaneous detection of the hydrodynamic size of the components even at very low concentrations (Fig. 5h; Supplementary Fig. 17)^46^. The purified samples containing only the stomatocytes entrapping nanoparticles (Fig. 5h, bottom line) were then analysed by ICP-MS to determine the amount of platinum entrapped (Supplementary Methods; Supplementary Table 2). The results showed the presence of 2.43±0.11 mg of platinum per 10 mg polymer (1 ml) for stomatocytes entrapping 60 nm PtNP.

Furthermore, additional cryo-TEM and TEM measurements of stomatocytes entrapping nanoparticles in the magnetic field at different tilt angles provided further proof that the particles were entrapped inside of the structures (Fig. 5g; Supplementary Figs 18–23).

In summary, we have shown the remote and fully reversible entrapment and release of cargo from stomatocytes via magnetic deformation. This is to our knowledge the first example of bowl-shaped structures that function as reversible supramolecular magneto-valves and it provides a new concept of magnetic manipulation of diamagnetic materials. The gradual increase in the size of the opening, which starts at fields lower than 10 T, opens the possibility of using these structures for delivery and nanoreactor applications. The use of such structures for delivery applications and magnetic resonance imaging, however, requires lower magnetic fields. Nevertheless, current modern clinical MRI systems are employing now much higher fields some operating at 3–7 T and even at 10 T. Such high fields raises questions regarding their potential physiological effects on humans, although recent studies showed no evidence of safety issues when using high field and even gradients^47^. However, since most of the routine MRI’s operates at lower fields and these high-field clinical instruments are still under scrutiny and debate for safe use in humans, the immediate medical applicability of our stomatocyte valve system is as for now limited. Our concept is in its infancy and is taking advantage of the stomatocyte double-compartmentalized morphology and its responsiveness in the magnetic field. This dual-compartmental design of the stomatocyte can introduce a second mechanism of cargo release in addition to the traditional release mechanisms specific to polymersomes, that is, by chemically changing the permeability of the membrane under a non-magnetic field stimulus. Finally, we have demonstrated that seemingly non-magnetic molecules can be made responsive in magnetic fields to generate dynamic functional assemblies opening more possibilities for a large variety of diamagnetic materials. Future work will focus on the manipulation with magnetic fields of other diamagnetic materials, as well as on designing polymers with higher magnetic susceptibility where this new concept can have a great potential for delivery applications at much lower fields, in particular, when the structures are responsive to multiple stimuli and not only to magnetic field.

## Methods

All reagents and chemicals were used as received unless otherwise indicated. Styrene was distilled before polymerization to remove the inhibitor. Anisole and *N*,*N*,*N*′,*N*′′,*N*′′-pentamethyl-diethylenetriamine were distilled before use. Ultrapure MilliQ water obtained with a Labconco Water Pro PS purification system (18.2 MΩ) was used for self-assembly of polymersomes and dialysis. Spectra/Por Dialysis Membrane MWCO: 12–14,000 g mol^−1^ was used for dialysis of polymersomes and their shape transformation in stomatocytes. Polyvinylpyrrolidone (Mn~10,000 g mol^−1^) and potassium tetrachloroplatinate (II) 99.9% were purchased from Sigma-Aldrich. L (+) ascorbic acid was purchased and used as received from Acros Organics.

### Synthesis of the amphiphile

The amphiphilic block-copolymer poly(ethylene glycol)_44_-*b*-polystyrene_177_ was synthesized by atom-transfer living radical polymerization via a previously reported procedure^31^. The size of the hydrophobic polystyrene block was adjusted to be in the range of 175 monomers for efficient polymersome formation. The block-copolymer was characterized by ^1^H NMR and gel permeation chromatography to evaluate the molecular weight and the size distribution. The sample showed narrow size distribution with a polydispersity index of 1.05.

### Preparation of PVP-capped PtNPs

Poly(vinylpyrrolidone) (PVP)-capped PtNPs with sizes ranging from 40 to 80 nm were synthesized via a modified sonication technique providing large concentration of nanoparticles with a tailored size depending on the concentration of the platinum salt, the concentration of the capping agent, as well as the sonication time and the time allowed for nucleation and growth^34^.

### Preparation of glassy stomatocytes

Rigid stomatocytes were prepared via a modified dialysis-induced shape transformation as reported previously^34^. The block-copolymer poly(ethylene glycol)_44_-*b*-polystyrene_177_ (20 mg) was dissolved in a 2-ml mixture of THF/dioxane (1.6 ml THF amd 0.4 ml dioxane) in a 15-ml capped vial equipped with a magnetic stirrer and closed with a rubber septum. After 30 min of stirring at room temperature, 2 ml of MilliQ water (with vigorous stirring, 900 r.p.m.) was added to the organic phase via a syringe pump using a 5-ml syringe equipped with a steel needle. The syringe pump was set up for an addition rate of 1 ml h^−1^. The solution turned cloudy after the addition of 0.5 ml of water. After complete addition of water the colloidal mixture was transferred in a dialysis bag and dialyzed against water for 48 h to form glassy rigid stomatocytes (final concentration 10 mg ml^−1^).

### Preparation of Pt-filled stomatocytes

PtNP-filled stomatocytes were prepared according to a previously reported procedure^34^. Poly(ethylene glycol)_44_-*b*-polystyrene_177_ (20 mg) was dissolved in a mixture of THF and 1,4-dioxane (2 ml, 1.6/0.4 ratio) in a 15-ml capped vial equipped with a magnetic stirrer and closed with a rubber septum. The solution was stirred (rate: 400 r.p.m.) for 30 min at room temperature to allow complete dissolution of the polymer. MilliQ (0.7 ml) water followed by 1.3 ml of different size PVP-capped PtNP (50–80) solution was then added to the organic phase with vigorous stirring (900 r.p.m.) via a syringe pump using a 5-ml syringe equipped with a steel needle. The syringe pump was set up for an addition rate of 1 ml h^−1^. The solution turned cloudy after the addition of the water (0.7 ml), indicating that polymersomes were already formed. After complete addition of the aqueous solution of PtNPs (1.3 ml), the colloidal mixture was transferred in a dialysis bag and dialysed against water. The dialysis water was replaced after 1 h followed by frequent changes for 48 h.

### Preparation of flexible stomatocytes

One ml of glassy stomatocytes solution (concentration 10 mg ml^−1^) was reverse dialysed for 20 min in a mixture of water: THF: dioxane (1:0.8:0.2) in a dialysis bag from Spectrum labs, model Spectrapor 4, 12–14 kD, diameter 6.4 mm. Once the dialysis was finished, the sample was used immediately for the magnetic deformation experiments. TEM analysis of the flexible stomatocytes did not show any change in the morphology and shape after the dialysis and the structures maintained their shape and size as before exposure to organic solvent (main text Fig. 2d–g).

### Capture and release of load via magnetic deformations

*Capture of PtNP in high magnetic field.* Flexible stomatocytes (50 μl, 10 mg ml^−1^) obtained after 20 min reverse dialysis were immediately placed in a quartz cuvette. Separately, 100 μl of a solution containing PVP-coated PtNP^31^ of approximate sizes of 40, 60 and 80 nm were reverse dialysed under the same conditions, in a mixture of water: THF: dioxane (1:0.8:0.2) to ensure the same composition of the dispersing solution and to avoid the quenching of the flexible stomatocytes upon addition. The flexible stomatocytes were placed in the magnet and the solution was swept from 0 to 20 T with a sweeping rate of 40 mT s^−1^. The solution was kept at 20 T for 10 s, after which the nanoparticle solution was added to the mixture. After an additional 60 s, the magnetic field strength was brought back to 0 T and the sample was quenched at 0 T with water. The stomatocytes capturing PtNPs were purified from the free platinum by spin filtration using spin filters (0.22 μm Millipore filters). The stomatocytes/platinum solution (300 μl × 2) was centrifuged for 1 min at 3,000 r.p.m. in an Eppendorf Centrifuge (5430 R). Subsequently, each concentrated sample was dispensed in MilliQ (300 μl) and the spin filtration was repeated five times; the purity of the stomatocytes from the free platinum was checked then using AF4-UV-Quels technique.

*Platinum encapsulation efficiency via ICP-MS analysis*. Pure stomatocytes capturing 60 nm platinum solutions (10 μl × 3, 20 μl × 3, 30 μl × 3, 40 μl × 3) were added into nitric acid (65%, 0.5 ml) and stirred for 3 h at 80 °C to destruct the polymeric vesicles. The total volume of every sample was adjusted to 5.0 ml MilliQ. The platinum-counts of each sample was standardized using internal standard counts. The ICP-MS measurements was performed on a Thermo Fischer Scientific X series I quadrupole machine. The sample was calculated to entrap 2.43±0.11 mg of Pt per 10 mg stomatoytes (1 ml).

*Release of PtNPs in high magnetic field*. A solution (100 μl) of flexible stomatocytes filled with 80 nm PtNP was placed in a quartz cuvette and exposed to a magnetic field of 20 T with a sweeping rate of 40 mT s^−1^. The samples were quenched in the magnet center at 20 T via 1.5 ml water addition using a plastic transfer tube. The quenched samples were further analysed by *ex situ* TEM and DLS.

*LB measurements*. Magnetic LB was measured in a 20-T Duplex Bitter magnet using a standard polarization modulation technique^48^. A HeNe laser was used (1.5 mW, 632.8 nm) to probe the dispersion contained inside a 5-mm-thick optical cell (Hellma) within a temperature-controlled environment at 23.0±0.1 °C. The LB was measured by sweeping the magnetic field between 0 and 20 T, in which a positive LB sign corresponds to a higher refractive index (or polarizability) parallel to the magnetic field compared with the perpendicular refractive index. See Supplementary Fig. 2 for a sketch of the LB setup.

## Additional information

**How to cite this article**: van Rhee, P. G. *et al.* Polymersome magneto-valves for reversible capture and release of nanoparticles. *Nat. Commun.* 5:5010 doi: 10.1038/ncomms6010 (2014).

## Supplementary Material

Supplementary Information Supplementary Figures 1-23, Supplementary Tables 1-2, Supplementary Note 1, Supplementary Methods and Supplementary References.

## Figures and Tables

**Figure 1:**
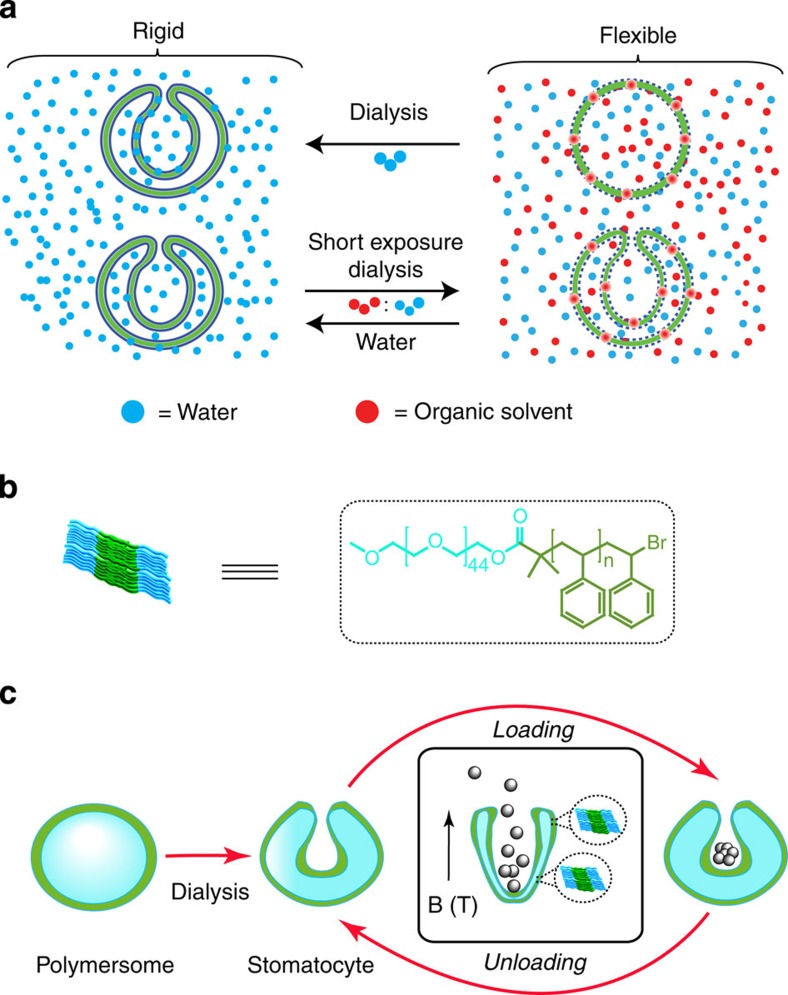
Reversible stomatocyte magnetic valve. (**a**) Schematic representation of stomatocyte formation and reversible change in its membrane flexibility from glassy to the plasticized state in the presence of organic solvent. The stomatocyte formation takes place under dialysis in pure water (blue spheres). The change in the flexibility of the membrane of stomatocytes from glassy to flexible takes place under reverse dialysis conditions in a mixture of water (blue spheres) and organic solvent (red spheres). (**b**) Representation of the amphiphilic block-copolymer assembly in a bilayer and its chemical structure. (**c**) Schematic representation of the reversible and controlled capture and release of particles under magnetic field via opening and closing of polymeric stomatocytes in the magnetic field. The stomatocytes were obtained by dialysis from diamagnetic polymersomes.
