# Supplementary material for: Polymersome magneto-valves for reversible capture and release of nanoparticles
Source: Nat Commun. 2014 Sep 24;5:5010. doi: 10.1038/ncomms6010 (PMC4176683; doi:10.1038/ncomms6010)
Supplement: Supplementary Information — Supplementary Figures 1-23, Supplementary Tables 1-2, Supplementary Note 1, Supplementary Methods and Supplementary References. [file ncomms6010-s1.pdf]

Supplementary Figures

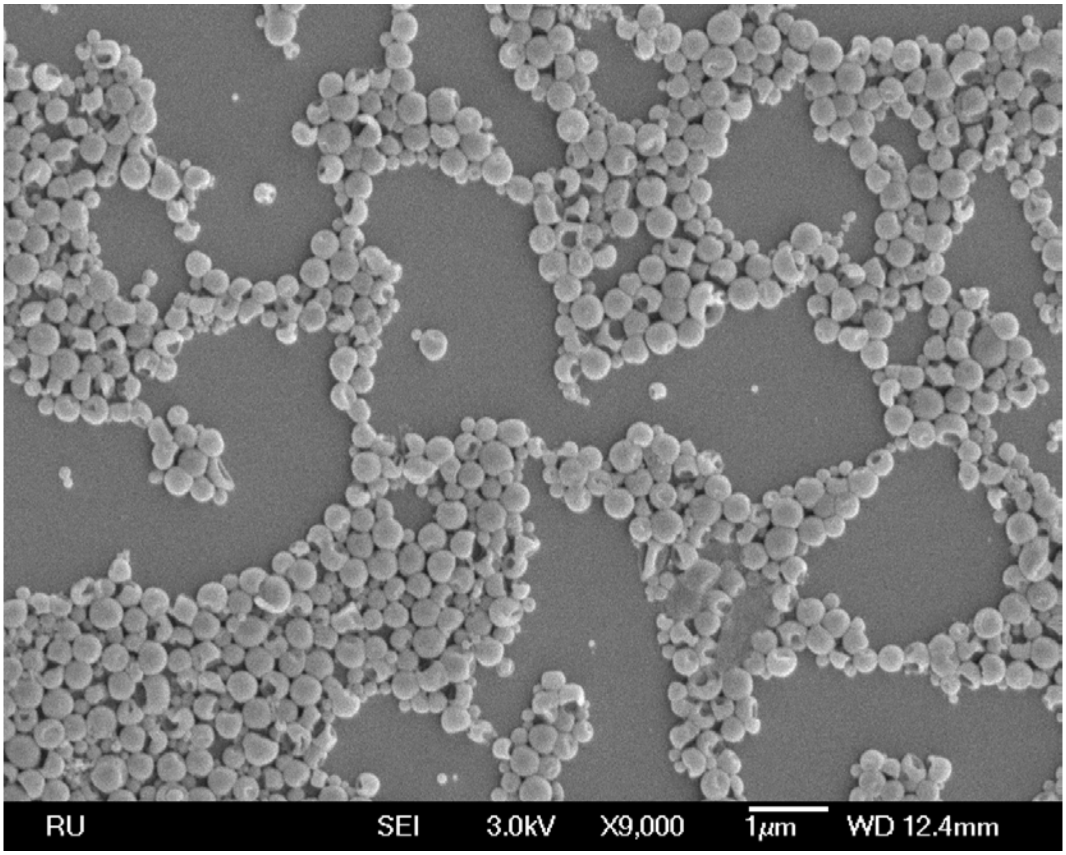

Supplementary Figure 1. Dry SEM images of stomatocytes after 20 min of reverse dialysis and quenching into water and before magnetic field exposure showing the same spherical shape of the stomatocyte as previous solvent exposure.

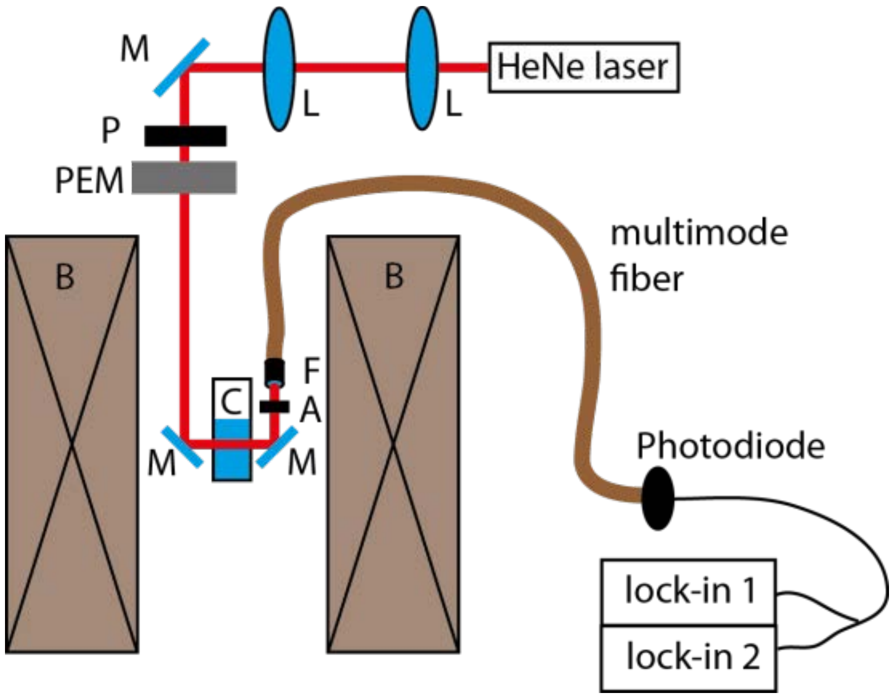

Supplementary Figure 2. Schematic representation of the linear birefringence setup. The laser light is transferred to a Si photodiode by a multimode fiber. A standard polarization modulation technique[1] was used which includes a photo-elastic modulator (PEM-90, Hinds Instruments) between crossed polarisers (P,A) to sensitively measure the LB at a high modulation frequency (50 kHz). An intensity-stabilised 632.8 nm HeNe laser was used (1.5 mW, Research Electro-Optics Inc) and the laser light (L) was guided by several mirrors. With two lenses ((L) focal lengths 50 & 200 mm) the laser beam was focused on the sample solution in a quartz cuvette ((C) Hellma, 5 mm), which was placed inside the temperature-controlled 32 mm bore of the 20 T Duplex Bitter magnet. The light was collected with a fiber lens (F) and a multimode fiber that guided the light to a silicon photodiode. The LB was determined using the first and second harmonic of the sinusoidal modulation, which was measured by two lock-in amplifiers (SR830, SRS). A positive sign of the LB corresponds to a parallel refractive index (or polarisability) that is higher than the perpendicular refractive index with respect to the magnetic field direction.

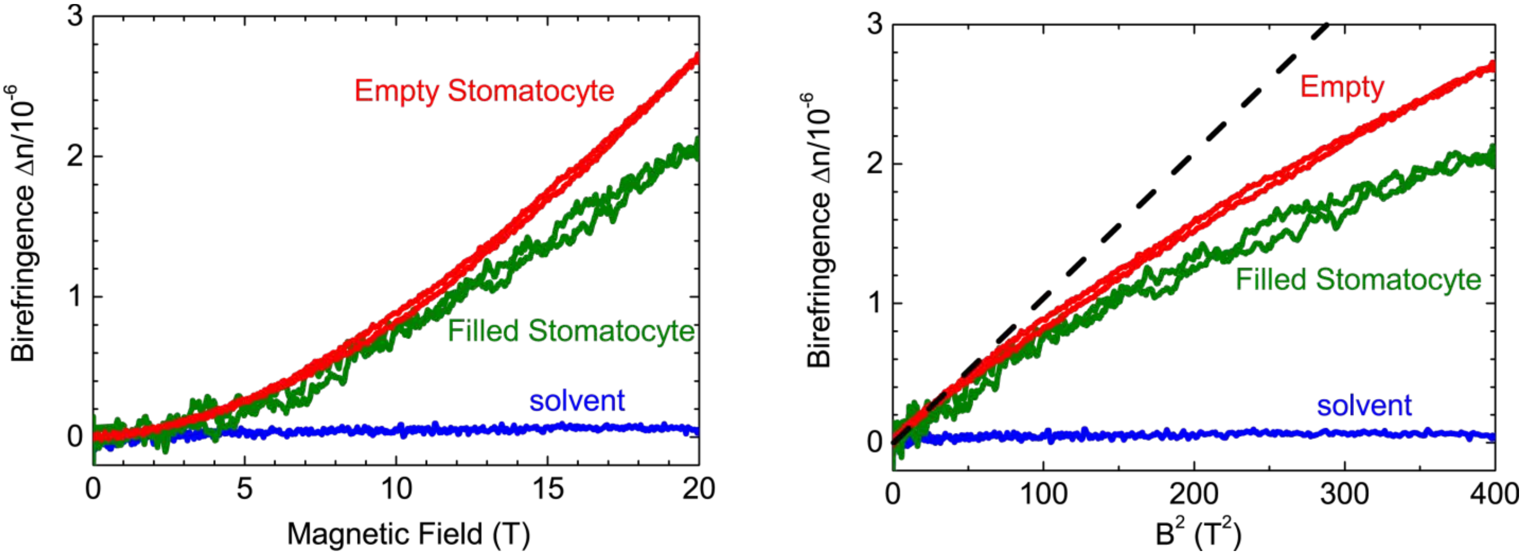

Supplementary Figure 3. *In situ* birefringence measurements of the flexible bowl-shaped stomatocyte filled with and without Pt nanoparticles. Shown are both up-field and down-field sweeps from 0 T to 20 T with 80 mT s^{-1} sweeping rate, respectively plotted against magnetic field B and the square of the magnetic field B^2.

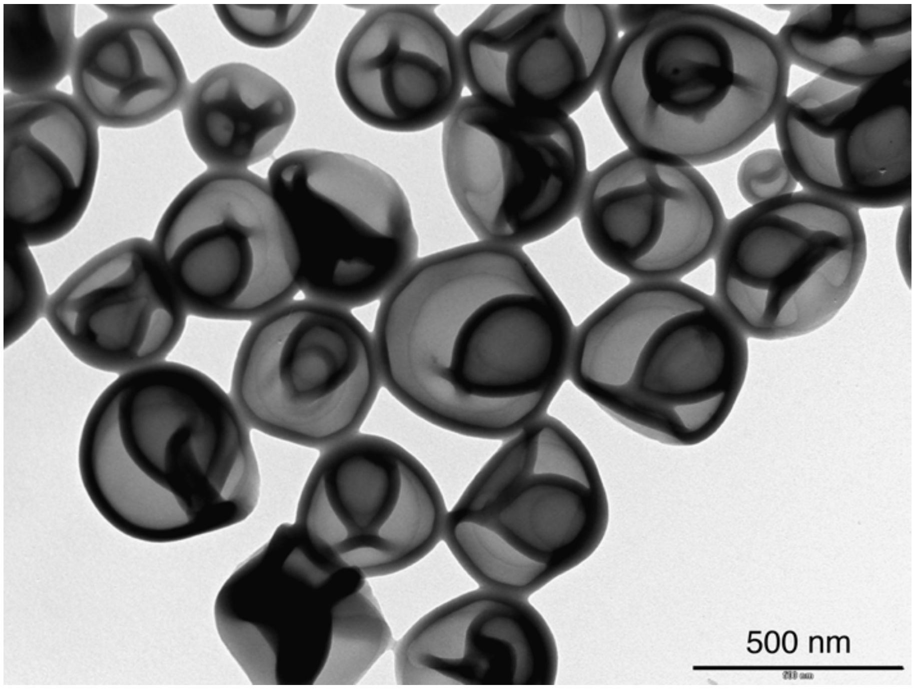

Supplementary Figure 4. Dry TEM of stomatocytes after 20 min of reverse dialysis and magnetic deformation 0 T – 20 T - 0 T and quenched at 0 T.

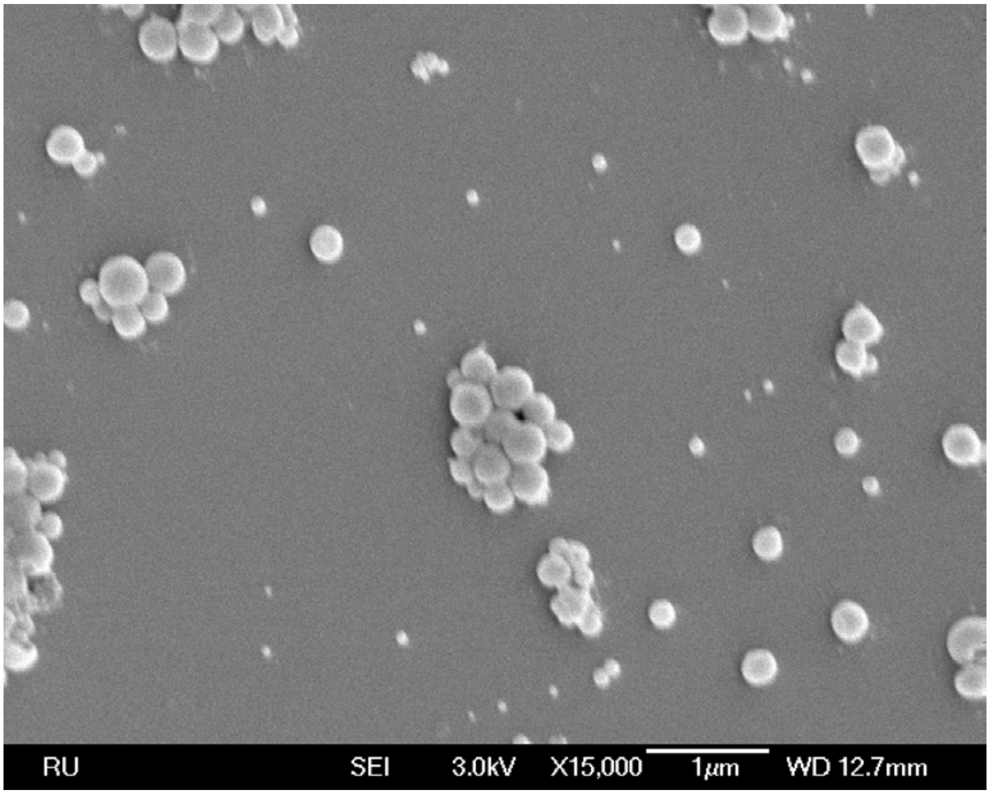

Supplementary Figure 5. Dry SEM images of stomatocytes after 20 min of reverse dialysis and magnetic deformation 0 T – 20 T - 0 T and quenched at 0 T showing the recovering of the overall spherical shape of the stomatocytes after exposure to the magnetic field.

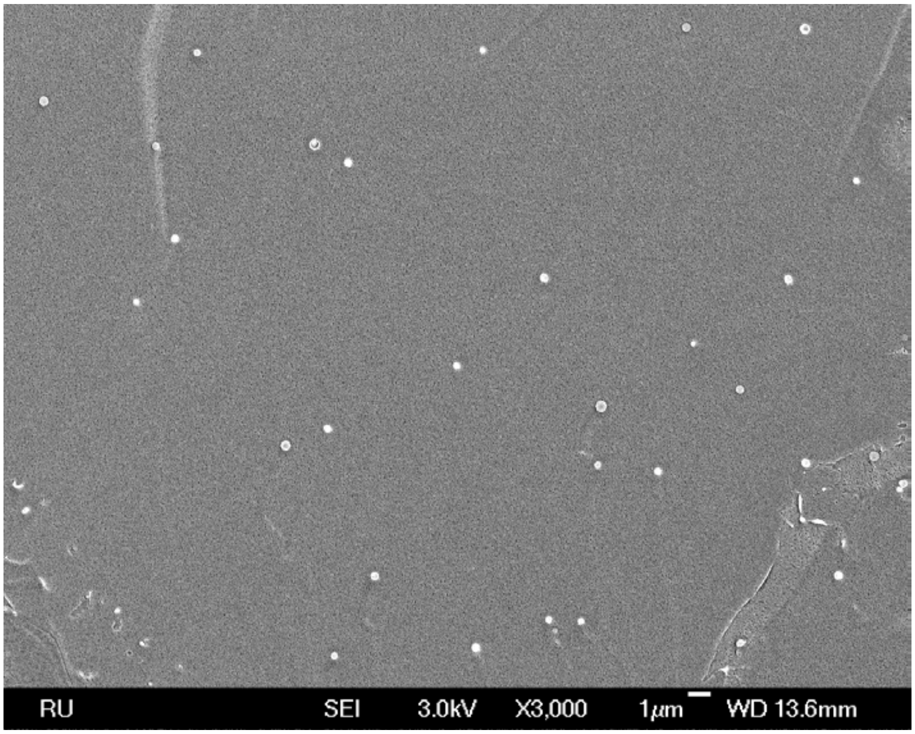

Supplementary Figure 6. Cryo-SEM images of stomatocytes after 20 min of reverse dialysis and magnetic deformation 0 T – 20 T - 0 T and quenched at 0 T showing the recovering of the overall spherical shape of the stomatocytes after exposure to the field.

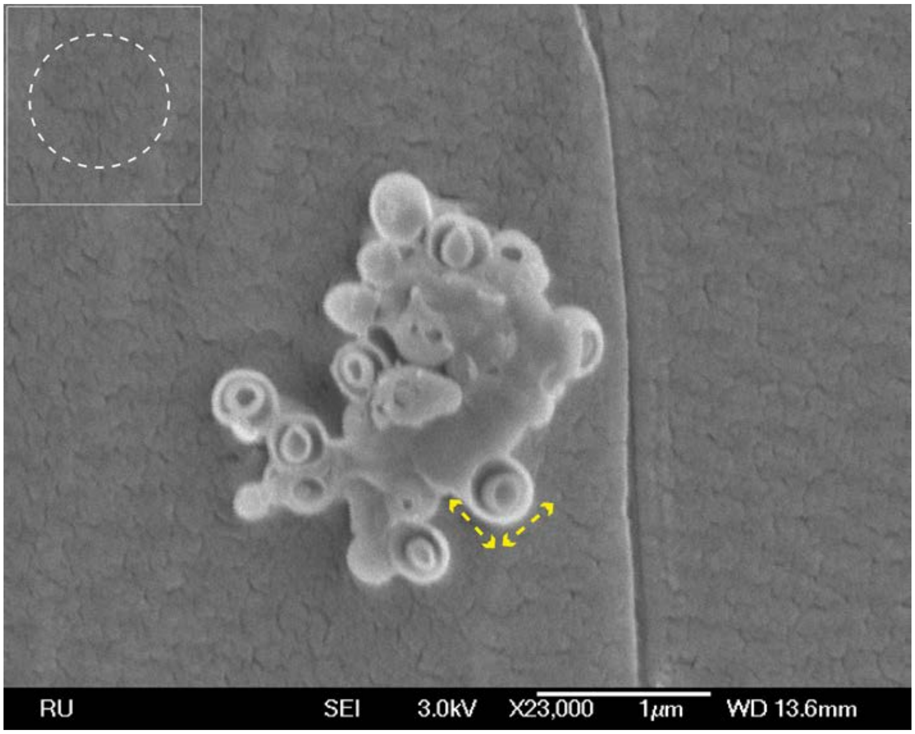

Supplementary Figure 7. Cryo-SEM images of stomatocytes after 20 min of reverse dialysis and magnetic deformation 0 T – 20 T - 0 T and quenched at 0 T showing the recovering of the overall spherical shape of the stomatocytes after exposure to the field. Note that these structures sectioned in half during the freeze fractioning SEM procedure allowing detail information of the inner structure of the stomatocytes and overall 3D spherical shape (inset).

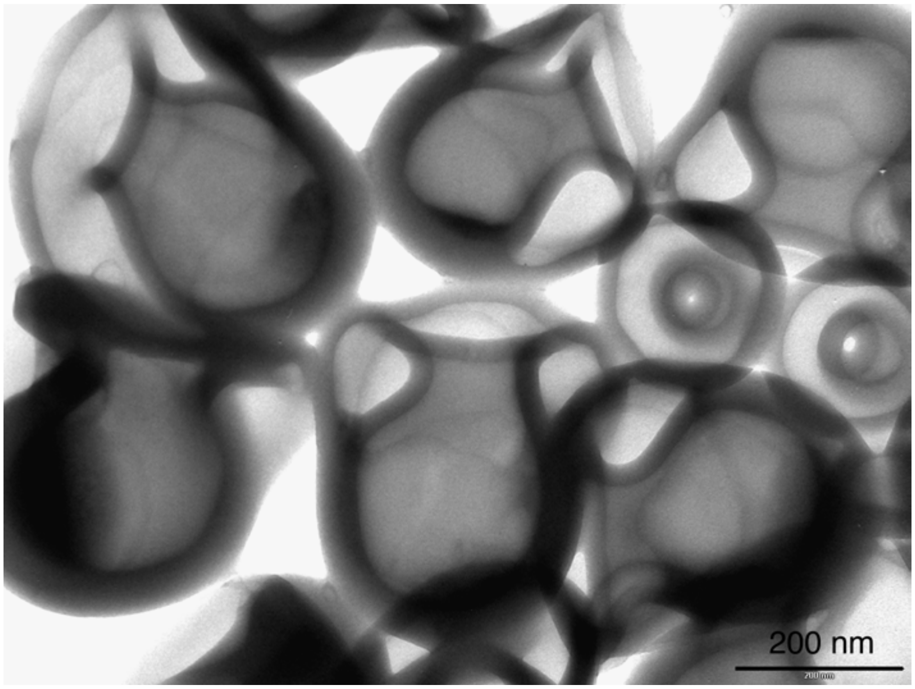

Supplementary Figure 8. Dry TEM images of stomatocytes after 20 min of reverse dialysis and magnetic deformation from 0 T - 20 T and quenched at 20 T showing the change in the overall shape of the stomatocytes into ellipsoidal structures with wide opening.

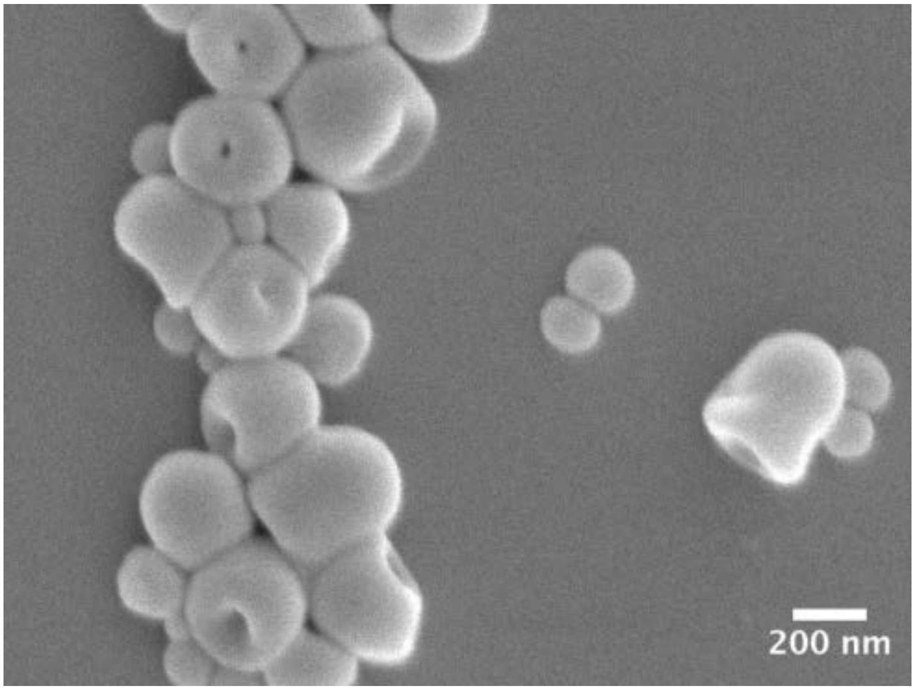

Supplementary Figure 9. Dry SEM images of stomatocytes after 20 min of reverse dialysis and magnetic deformation from 0 T - 20 T and quenched at 20 T showing the change in the overall shape of the stomatocytes into ellipsoidal structures with wide opening.

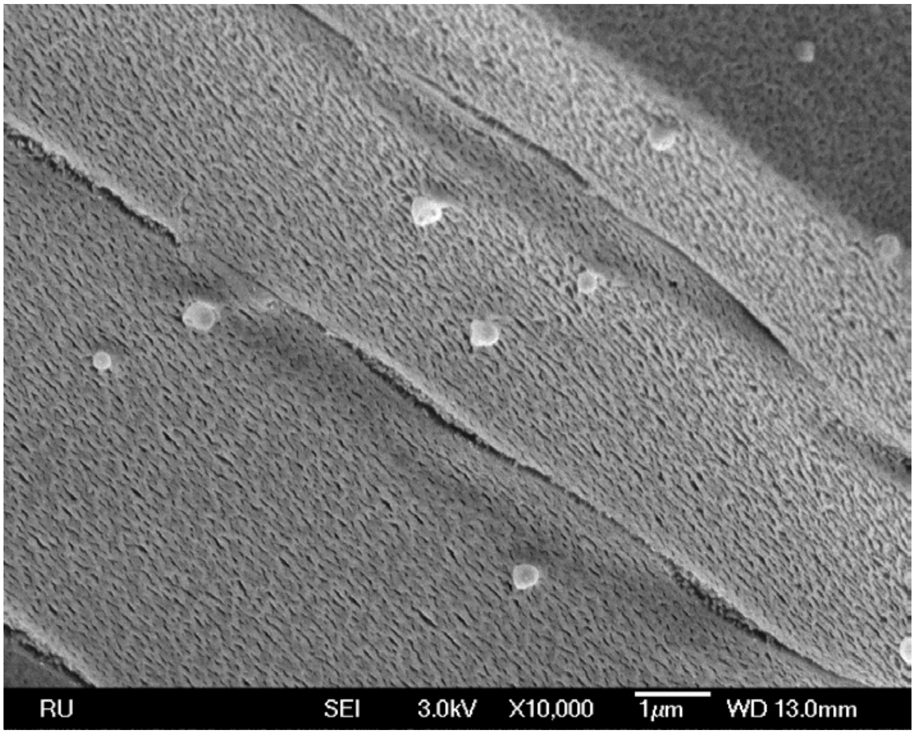

Supplementary Figure 10. Cryo-SEM images of stomatocytes after 20 min of reverse dialysis and magnetic deformation from 0 T - 20 T and quenched at 20 T showing the change in the overall shape of the stomatocytes into ellipsoidal structures with wide opening.

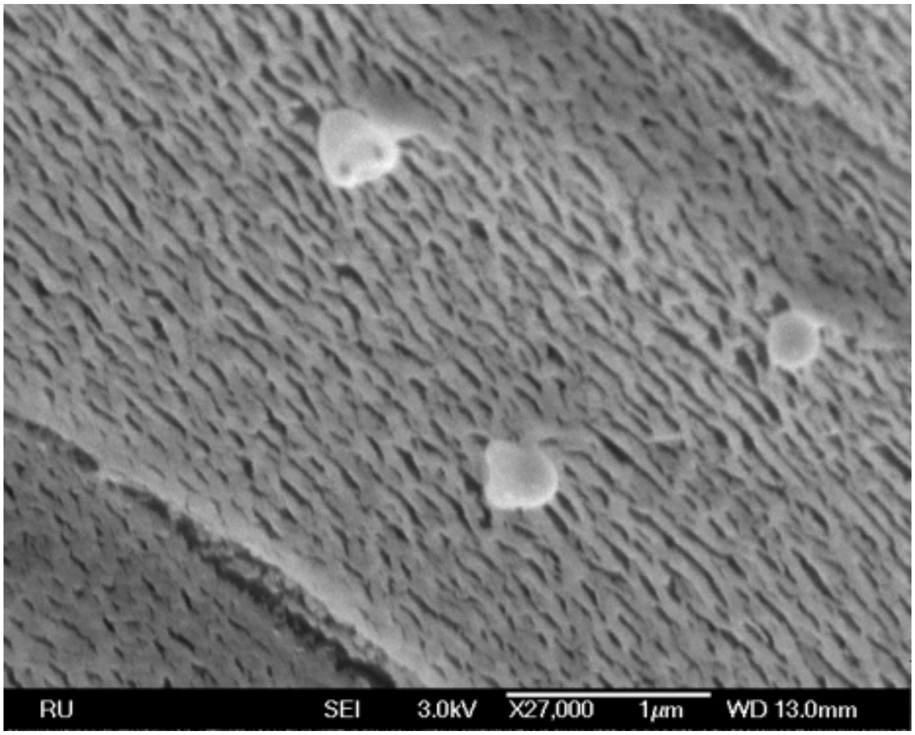

Supplementary Figure 11. Cryo-SEM images of stomatocytes after 20 min of reverse dialysis and magnetic deformation from 0 T - 20 T and quenched at 20 T showing the change in the overall shape of the stomatocytes into ellipsoidal structures with wide opening.

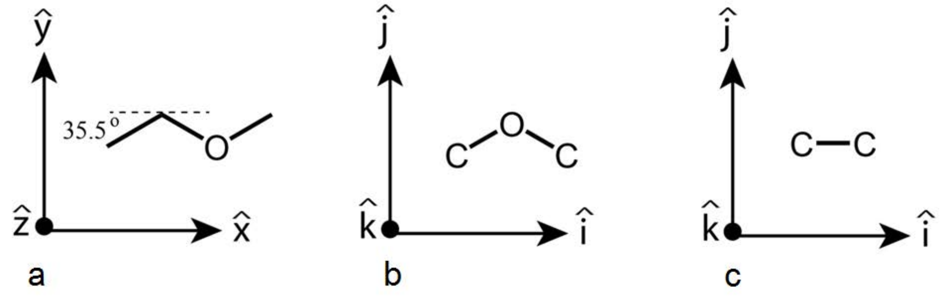

Supplementary Figure 12. a, A PEG monomer in the lab frame. **b**, The C-O-C group in its molecular frame. **c**, The C-C bond in its molecular frame.

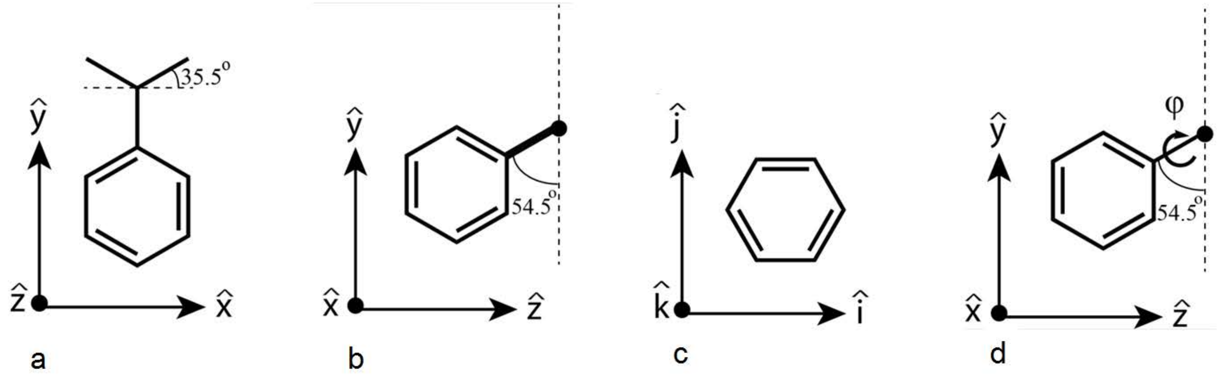

Supplementary Figure 13. a, A PS monomer in the lab frame. **b**, The C-C bond connecting the phenyl group to the backbone in the lab-frame. **c**, Phenyl ring in its molecular frame. **d,** The phenyl ring can rotate around the C-C bond connecting it to the backbone. Its orientation is defined by angle φ.

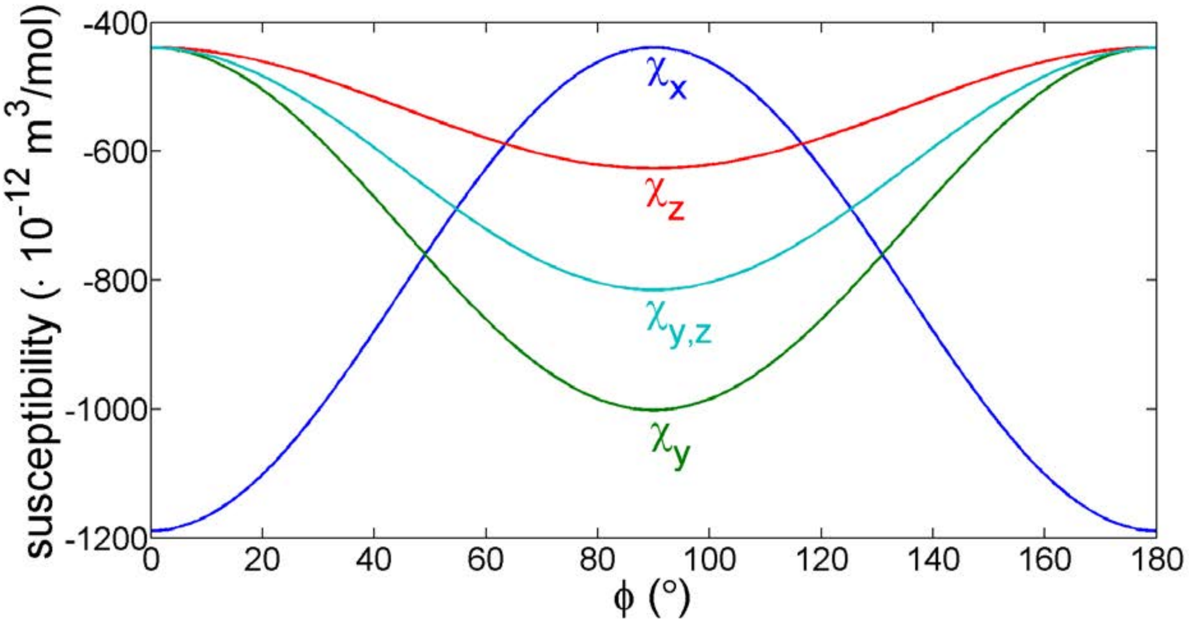

Supplementary Figure 14. Contribution of the phenyl group to the diamagnetic susceptibility in the x, y, z or axis of the PS molecule as function of the angle φ. The average value of χ_y and χ_z is also given as $\chi_{y,z}$.

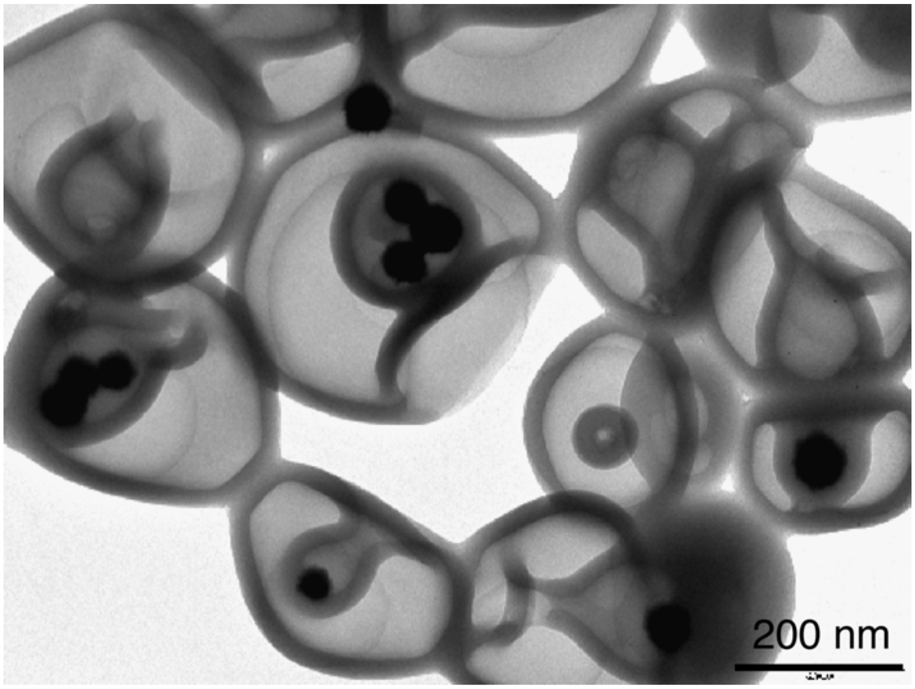

Supplementary Figure 15. TEM of PtNP-filled stomatocytes before magnetic field exposure.

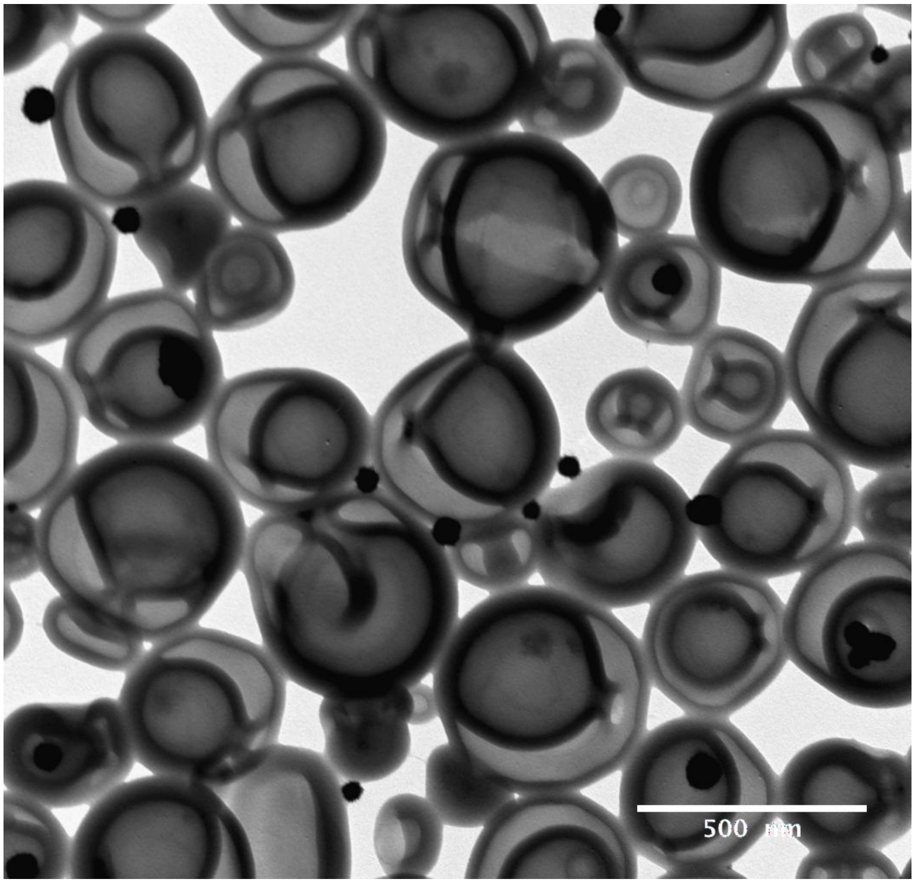

Supplementary Figure 16. TEM of PtNP-filled stomatocytes after exposure to magnetic field where deformation and release at 20 T occurs and return to the 0T field. The samples were then quenched in the magnet at 0 T with water. Note the recovering of the overall spherical shape after field exposure and the release of the PtNP outside of the stomatocyte inner cavity

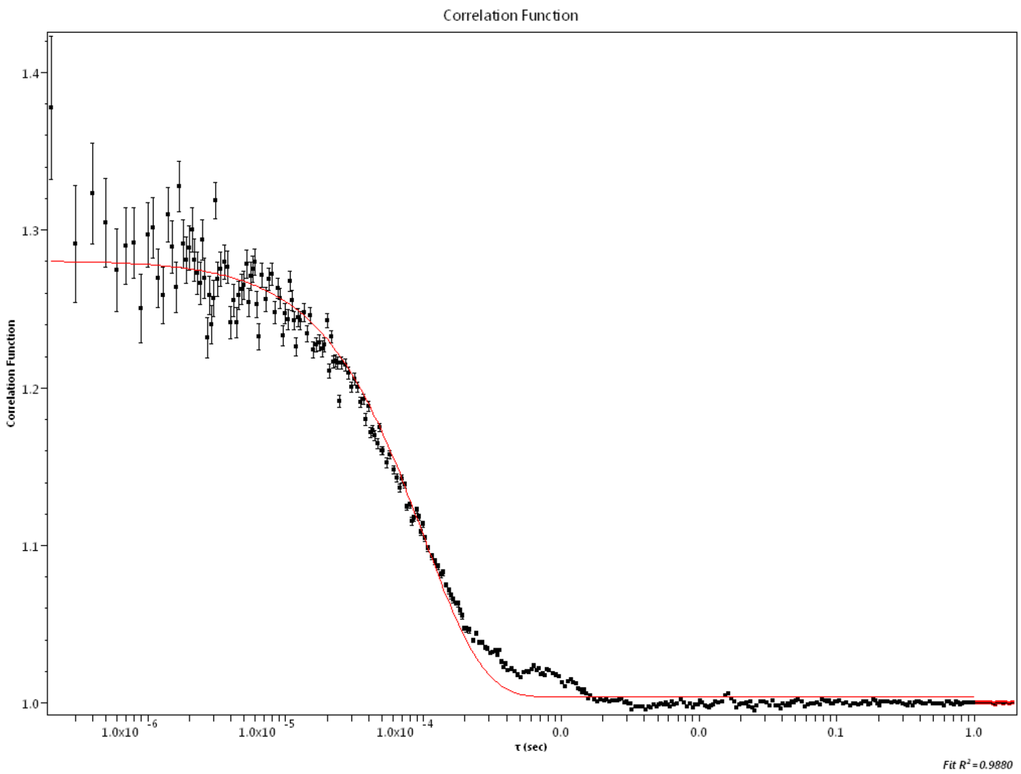

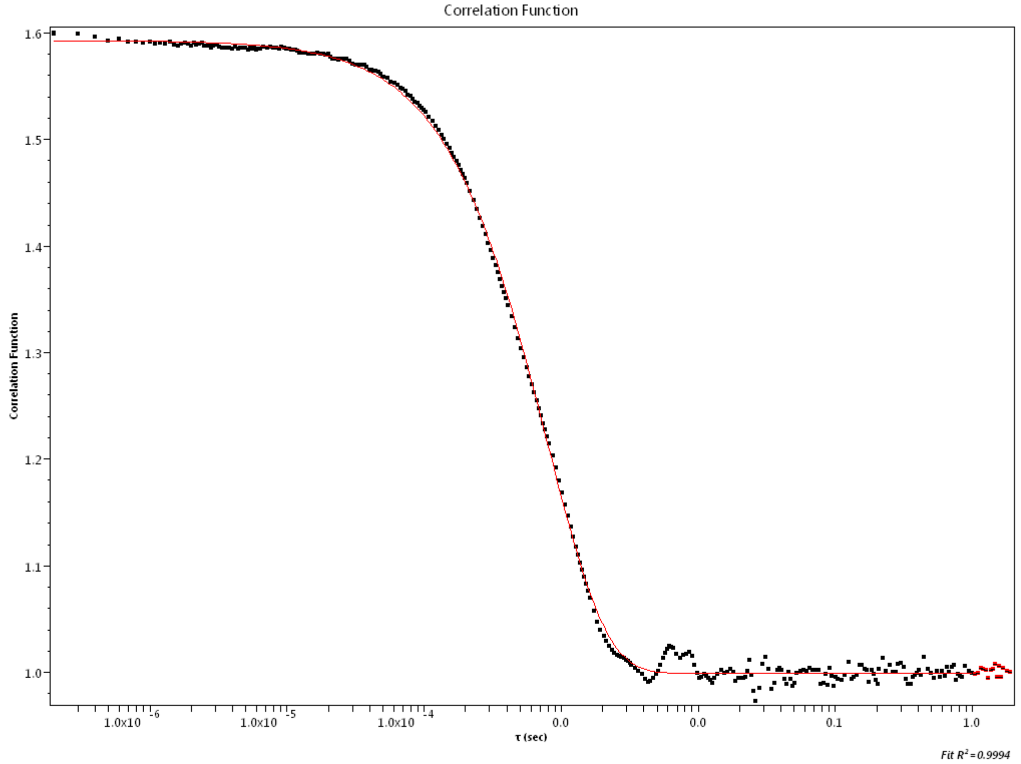

Supplementary Figure 17. (Top) Correlation function from the top of the fraction peak of platinum nanoparticles and is corresponding to a hydrodynamic radius of 31.90 nm. (Right) Correlation function from the top of the fraction peak of the stomatocytes capturing platinum nano-particles and is corresponding to a hydrodynamic radius of 255.97 nm.

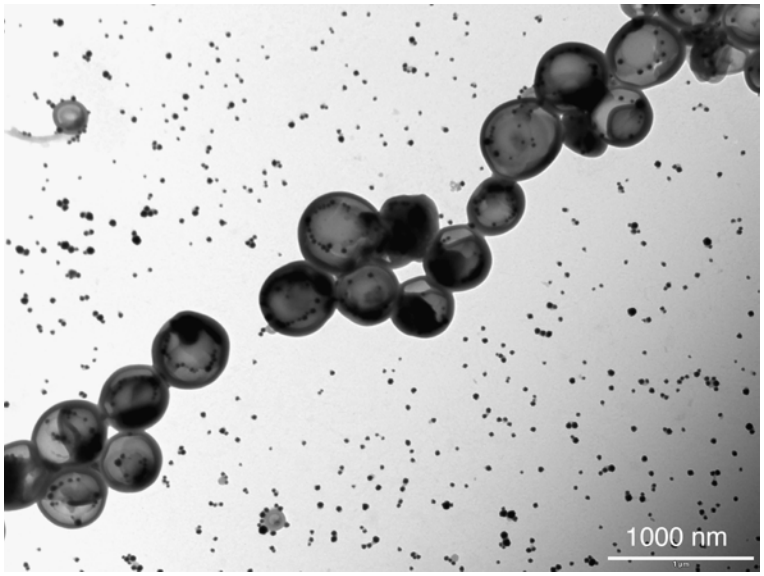

Supplementary Figure 18. TEM of PtNP-filled stomatocytes captured after opening the stomatocytes at 20 T and closing them at 0 T. The samples were then quenched in the magnet at 0 T with water. Note the presence of the ptNP inside of the stomach, with nanoparticles adhering to the inner cavity of the stomatocytes.

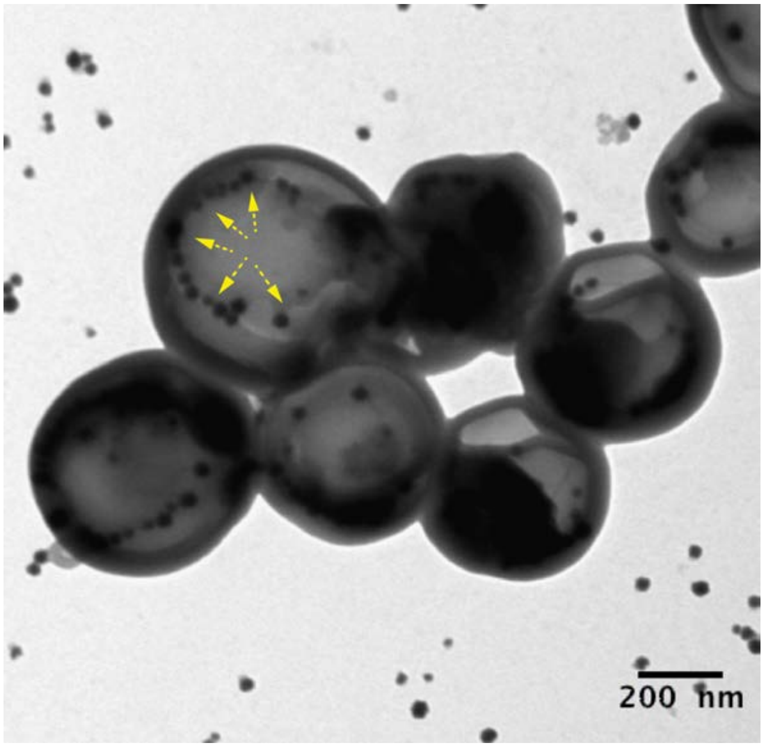

Supplementary Figure 19. TEM of PtNP-filled stomatocytes captured after opening the stomatocytes at 20 T and closing them at 0 T. The samples were then quenched in the magnet at 0 T with water. Note the presence of the ptNP inside of the stomach, with nanoparticles adhering to the inner cavity of the stomatocytes. If not entrapped, the particles would have been randomly distributed all over the structures and also on the membrane and not in a circular pattern inside the stomatocytes.

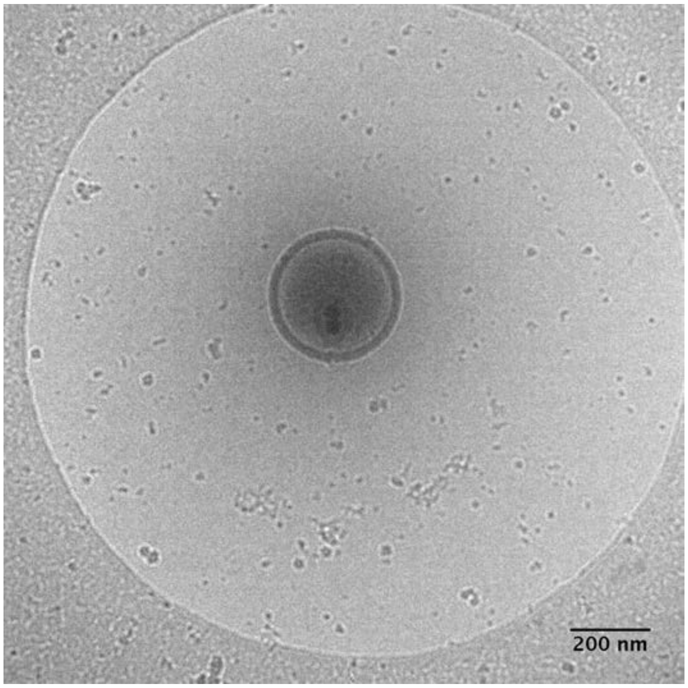

Supplementary Figure 20. Cryo-TEM of PtNP-filled stomatocytes captured after opening the stomatocytes at 20 T and closing them at 0 T. The samples were then quenched in the magnet at 0 T with water. The TEM grid was tilted at different rotation angles to give details of the localization of the platinum stomatocytes. The image is shown at zero rotation. The platinum nanoparticles are clearly seen entrapped inside of the structures.

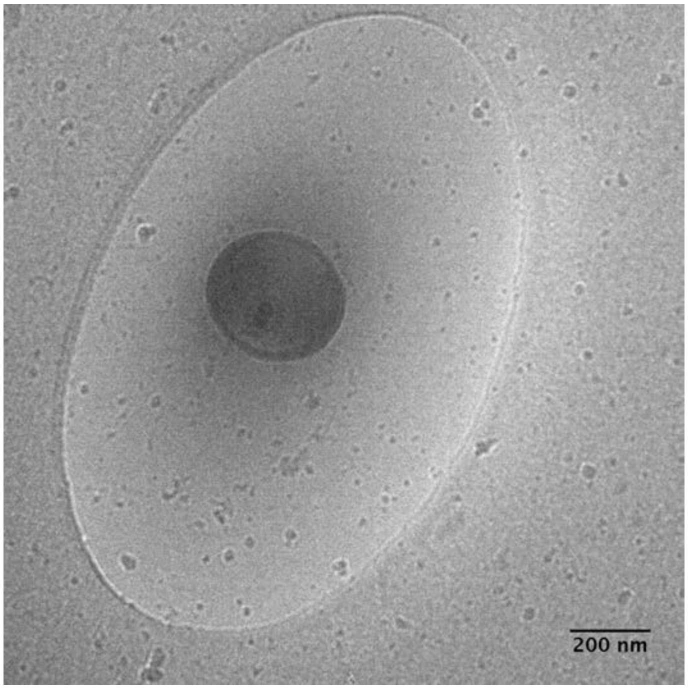

Supplementary Figure 21. Cryo-TEM of PtNP-filled stomatocytes captured after opening the stomatocytes at 20 T and closing them at 0 T. The samples were then quenched in the magnet at 0 T with water. The TEM grid was tilted at different rotation

angles to give details of the localization of the platinum stomatocytes. The image is shown at -45 deg rotation. The platinum nanoparticles are clearly seen entrapped inside of the structures.

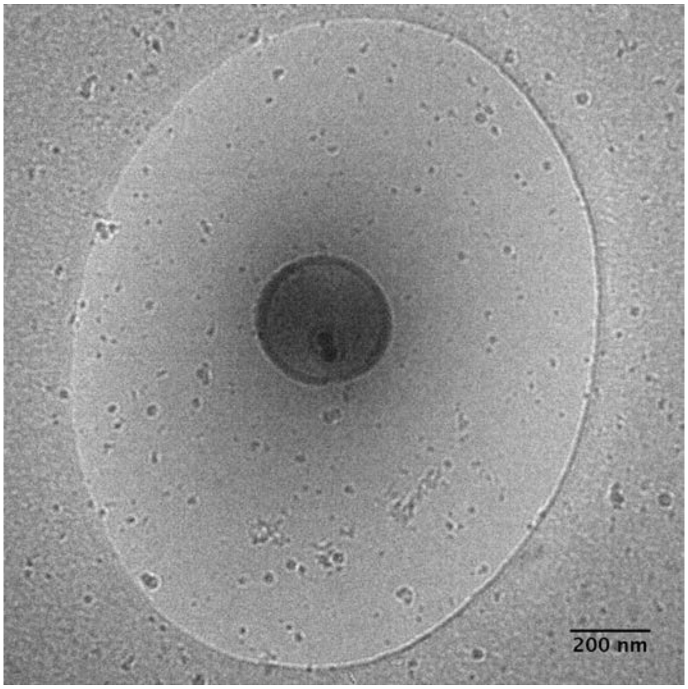

Supplementary Figure 22. Cryo-TEM of PtNP-filled stomatocytes captured after opening the stomatocytes at 20 T and closing them at 0 T. The samples were then quenched in the magnet at 0 T with water. The TEM grid was tilted at different rotation angles to give details of the localization of the platinum stomatocytes. The image is shown at +45 deg rotation. The platinum nanoparticles are clearly seen entrapped inside of the structures.

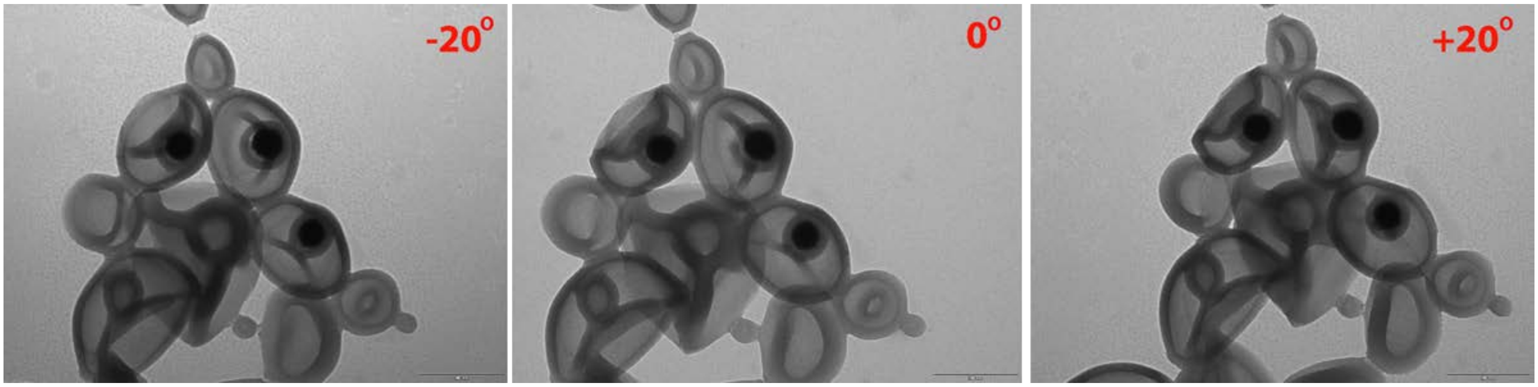

Supplementary Figure 23. TEM of PtNP-filled stomatocytes captured after opening the stomatocytes at 20 T and closing them at 0 T. The samples were then quenched in the magnet at 0 T with water. The TEM grid was tilted at different rotation angles to give details of the localization of the platinum stomatocytes. The platinum nanoparticles are clearly seen entrapped inside of the structures.

Supplementary Tables

Start (min)	End (min)	Mode	Cross flow start (mL min^{-1})	Cross flow end (mL min^{-1})
0	1	Elution	3.00	3.00
1	2	Focus	-	-
2	3	Focus + inject	-	-
3	4	Focus	-	-
4	6	Elution	3.00	1.17
6	8	Elution	1.17	0.49
8	10	Elution	0.49	0.24
10	13	Elution	0.10	0.10
13	30	Elution	0.10	0.10
30	31	Elution	0.00	0.00
31	32	Elution + inject	0.00	0.00
32	37	Elution	0.00	0.00

Supplementary Table 1: General method for the FFF separation. The following conditions were used for the FFF separation: 1.0 mL min^{-1} detector flow, 1.00 mL min^{-1} focus flow and 0.20 injection flow. The hydrodynamic radii from the Quels (DLS) of the purified sample (the captureof PtNP experiments for the quantification experiments were the following: Platinum nano-particles: 32.9 nm (±0.4 %), Stomatocytes capturing platinum nano-particles: 254.6 (±0.8 %).

Sample	Dilution Factor	Measured Pt concentration ppb	Calculated Pt concentration in the undiluted sample (mg mL^{-1})
1	500	4932	2.466
2	500	4844	2.422
3	500	4614	2.307
4	250	9752	2.438
5	250	9863	2.466
6	250	10060	2.515
7	166.67	14190	2.365
8	166.67	12950	2.158
9	166.67	15190	2.532
10	125	20460	2.558
11	125	19700	2.463
12	125	19620	2.453

Supplementary Table 2: Platinum encapsulation efficiency via ICP-MS analysis. Pure stomatocytes capturing 60 nm platinum solutions (10 µL x 3, 20 µL x 3, 30 µL x 3, 40 µL x 3) were added into nitric acid (65 %, 0.5 mL) and stirred for 3 hours at 80° C to destruct the polymeric vesicles. The total volume of every sample was adjusted to 5.0 mL MilliQ. The platinum-counts of each sample was standardized using internal standard counts. The inductively coupled plasma – mass spectroscopy (ICP-MS) measurements was performed on a Thermo Fischer Scientific X series I quadrupole machine. The sample was calculated to entrap 2.43 ± 0.11 mg of Pt / 10 mg stomatocytes (1 mL)

Supplementary Note 1 Diamagnetic anisotropy of poly(ethylene glycol)-polystyrene

Diamagnetic anisotropy of PEG In this subsection the anisotropy of the magnetic susceptibility $\Delta\chi$ is calculated for fully extended PEG and PS polymers. Since in reality the polymers in the membrane are coiled to some extent, the actual anisotropy will be lower than the value calculated here which represents the maximal $\Delta\chi$ possible. However, the calculation presented is only meant to give insight into the sign of the magnetic anisotropy, which will be identical for both fully extended and partly coiled polymers. In Supplementary Fig. 12a, one monomer of PEG is shown. This monomer consists of one C-O-C and one C-C group as shown in Supplementary Fig. 12b,c. The diamagnetic anisotropy of these groups (in 10^{-12} m^3 mol^{-1}) are:

C-O-C[4]: C-C[5]:

$$\chi_i^{(COC)} = \chi_i^{(COC)} \qquad \chi_i^{(CC)} = \chi_i^{(CC)}$$
$$\chi_j^{(COC)} = \chi_i^{(COC)} + 49 \qquad \chi_j^{(CC)} = \chi_i^{(CC)} + 16 \qquad (1)$$
$$\chi_k^{(COC)} = \chi_i^{(COC)} + 82 \qquad \chi_k^{(CC)} = \chi_i^{(CC)} + 16$$

The C-O-C frame (i,j,k) coincides with the lab frame of the polymer (x,y,z) and therefore:

$$\chi_x^{(COC)} = \chi_i^{(COC)} \qquad \chi_y^{(COC)} = \chi_j^{(COC)} \qquad \chi_z^{(COC)} = \chi_k^{(COC)} \qquad (2)$$

The i-axis of the C-C frame (i,j,k) makes an angle of 35.5° with respect to the x-axis of the polymer frame (x,y,z) and therefore:

$$\chi_x^{(CC)} = \chi_i^{(CC)} \cdot \cos^2(35.5) + \chi_j^{(CC)} \cdot \sin^2(35.5) = \chi_i^{(CC)} + 5.4$$
$$\chi_y^{(CC)} = \chi_i^{(CC)} \cdot \sin^2(35.5) + \chi_j^{(CC)} \cdot \cos^2(35.5) = \chi_i^{(CC)} + 10.6 \qquad (3)$$
$$\chi_z^{(CC)} = \chi_k^{(CC)} = \chi_i^{(CC)} + 16$$

This gives a total diamagnetic susceptibility per monomer of PEG of:

$$\chi_x^{(PEG)} = \chi_i^{(COC)} + \chi_i^{(CC)} + 5.4$$
$$\chi_y^{(PEG)} = \chi_i^{(COC)} + \chi_i^{(CC)} + 59.6 \qquad (4)$$
$$\chi_z^{(PEG)} = \chi_i^{(COC)} + \chi_i^{(CC)} + 98$$

Since only the absolute differences between x, y and z matter, we can write it as:

$$\chi_x^{(PEG)} = 5.4$$
$$\chi_y^{(PEG)} = 59.6 \qquad (5)$$
$$\chi_z^{(PEG)} = 98$$

Assuming that the polymers in the membrane are rotated along the molecular x-axis into random orientations, χ_y and χ_z will average out to one component perpendicular to χ_x:

$$\chi_x^{(PEG)} = 5.4$$

$$\chi_{y,z}^{(PEG)} = 78.8 \quad (6)$$

So $\Delta\chi^{(PEG)} = \chi_x^{(PEG)} - \chi_{y,z}^{(PEG)} = -73.4$ ($\cdot\ 10^{-12}$ m^3 mol^{-1}).

Diamagnetic anisotropy of atactic polystyrene. In Supplementary Fig. 13a, one monomer of polystyrene is shown in the lab frame. This monomer consists of one phenyl group, two C-C bonds in the backbone and one C-C bond connecting the phenyl to the backbone.

We will consider the C-C bonds in the backbone first. The i-axis of the C-C frame (i,j,k) makes an angle of 35.5^o with respect to the x-axis of the polymer frame (x,y,z) for both C-C bonds in the backbone. The contribution of each of these two bonds to the magnetic susceptibility of the PS monomer in the frame of the polymer can be written as:

$$\chi_x^{(CC)} = \chi_i^{(CC)} + 5.4$$

$$\chi_y^{(CC)} = \chi_i^{(CC)} + 10.6 \quad (7)$$

$$\chi_z^{(CC)} = \chi_i^{(CC)} + 16$$

Now, we will consider the C-C bond connecting the phenyl group to the backbone (Supplementary Fig. 13b). The i-axis of the C-C frame (i,j,k) makes an angle of 54.5 ^{o} with respect to the y-axis of the polymer frame (x,y,z) and therefore:

$$\chi_x^{(CC)} = \chi_i^{(CC)} + 16$$

$$\chi_y^{(CC)} = \chi_i^{(CC)} \cdot \cos^2(54.5) + \chi_j^{(CC)} \cdot \sin^2(54.5) = \chi_i^{(CC)} + 10.6 \quad (8)$$

$$\chi_z^{(CC)} = \chi_i^{(CC)} \cdot \sin^2(54.5) + \chi_j^{(CC)} \cdot \cos^2(54.5) = \chi_i^{(CC)} + 5.4$$

The three C-C bonds in a PS monomer thus give:

$$\chi_x^{(allCC)} = 3 \cdot \chi_i^{(CC)} + 26.8$$

$$\chi_y^{(allCC)} = 3 \cdot \chi_i^{(CC)} + 31.8 \quad (9)$$

$$\chi_z^{(allCC)} = 3 \cdot \chi_i^{(CC)} + 37.4$$

Since only the absolute differences between x, y and z matter, we can write it as:

$$\chi_x^{(allCC)} = 26.8$$

$$\chi_{y,z}^{(allCC)} = 34.6 \quad (10)$$

so $\Delta\chi^{(allCC)} = -7.8$

Finally, the contribution of the phenyl group needs to be considered (Supplementary Fig. 13c). The diamagnetic anisotropy of a phenyl group (in 10^{-12} m^3 mol^{-1}) is:[4]

$$\chi_i^{(Ph)} = -439$$
$$\chi_j^{(Ph)} = -439 \qquad (11)$$
$$\chi_k^{(Ph)} = -1189$$

The i-axis of the phenyl frame makes an angle of 54.5° with respect to the y-axis of the polymer frame (x,y,z). Also, the phenyl group can rotate along the C-C bond that connects it to the backbone, thereby introducing an angle φ (with φ = 0 corresponding to the conformation as drawn in Supplementary Fig. 13d). The diamagnetic susceptibility of the phenyl group in the polymer frame can therefore be written as:

$$\chi_x^{(Ph)} = \chi_k^{(Ph)} \cdot \cos^2(\varphi) + \chi_j^{(Ph)} \cdot \sin^2(\varphi)$$
$$\chi_y^{(Ph)} = (\chi_k^{(Ph)} \cdot \sin^2(\varphi) + \chi_j^{(Ph)} \cdot \cos^2(\varphi)) \cdot \sin^2(\theta) + \chi_i^{(Ph)} \cdot \cos^2(\theta) \qquad (12)$$
$$\chi_z^{(Ph)} = (\chi_k^{(Ph)} \cdot \sin^2(\varphi) + \chi_j^{(Ph)} \cdot \cos^2(\varphi)) \cdot \cos^2(\theta) + \chi_i^{(Ph)} \cdot \sin^2(\theta)$$

In Supplementary Fig. 14, $\chi_x^{(Ph)}$, $\chi_y^{(Ph)}$, $\chi_z^{(Ph)}$ and $\chi_{y,z}^{(Ph)}$ are plotted as function of φ. $\chi_x^{(Ph)}$ is smaller than $\chi_{y,z}^{(Ph)}$ for angles of 0° to 54.7° and 125.3° to 180°. This means that in this region the contribution of the phenyl groups leads to a perpendicular alignment of the PS relative to the magnetic field. The angle φ (or possible angles φ) that the phenyl group can adopt determines the diamagnetic susceptibilities in the x, y and z directions. If the phenyl group is free to rotate, or if many phenyl rings adopt all possible orientations equally, the average contribution to the anisotropy of the diamagnetic susceptibility per phenyl is: $\Delta\chi^{(Ph)} = -188.3$ ($\cdot 10^{-12}$ m^3 mol^{-1}).

A free rotating phenyl group leads to a $\chi_x^{(Ph)}$ which is lower than $\chi_{y,z}^{(Ph)}$ which supports the idea that the polymer aligns perpendicular to the magnetic field. However, according to the literature[6-8], the phenyl group will not rotate freely. Atactic PS has its phenyl groups pointed randomly left or right of the polymer backbone. It can therefore consist of regions of isotactic and syndiotactic PS. According to Jasse and Koenig[6], stretched oriented polystyrene shows increasing trans C-C bonds at the expense of gauge bonds, so the syndiotactic polystyrene backbone can be best taken to estimate the maximal diamagnetic anisotropy. As for the angle of the phenyl group with respect to the backbone, Jasse and Koenig measured an angle of 35°, which was confirmed by Vancso *et al.*[7,8] who determined this angle to be between 30° – 40°. From Supplementary Fig. 14 it follows that each phenyl group will contribute to the diamagnetic anisotropy with a $\Delta\chi^{(Ph)} = -380.2$ ($\cdot 10^{-12}$ m^3 mol^{-1}). Adding to this the contribution of the backbone, we get: $\Delta\chi^{(PS)} = \Delta\chi^{(Ph)} + \Delta\chi^{(allCC)} = -388$ ($\cdot 10^{-12}$ m^3 mol^{-1}).

In conclusion the calculated $\Delta\chi$'s are for maximal extended polymers, so they represent the maximal $\Delta\chi$ possible. The phenyl groups as well as the PS and PEG backbones all contribute to a negative $\Delta\chi$, which means that the polymers will align perpendicular to an applied magnetic field, which is in full agreement with the experimental results. In reality, the polymer chain

will not be fully extended but rather be randomly coiled to some extent. This will decrease the absolute value of $\Delta\chi$. However, the sign of $\Delta\chi$ will remain unaffected.

Effect of the magnetic field on stomatocytes at different length scales Effects of the magnetic field on the structure of the stomatocyte can only be expected if the magnetic energy is in the order of kT. The difference in magnetic energy, ΔE, can be calculated with $\Delta E = \frac{-1}{2\mu_0 N_A}\Delta\chi B^2$, where B is the applied magnetic field, μ_0 the magnetic permeability of vacuum and $\Delta\chi$ the anisotropy of the magnetic susceptibility. For a single C-C bond at 22^{0}C and 20T, $\Delta E/kT = 1.04\cdot10^{-6}$. Since the magnetic energy is 6 orders of magnitude smaller than kT, no magnetic field effect can be expected on the scale of a single C-C bond. For a fully extended PEG_{44}-PS_{177} block-copolymer we calculate a $\Delta\chi$ of $-7.2\cdot10^{-8}$ m^3 mol^{-1}, which leads to a relative magnetic energy of $\Delta E/kT = 0.0047$. The magnetic energy of a single block-copolymer is still 2 to 3 orders of magnitude smaller than kT. Therefore, the degree of magnetic alignment of one single free block-copolymer is negligible. One needs a total of 213 fully extended polymers to obtain a magnetic energy equal to kT. This demonstrates that the effect of the magnetic field will only be noticeable if enough polymers are clustered together, like in the membrane of a stomatocyte.

Supplementary Method 1 Instrumentation.

Dynamic light scattering (DLS) experiments were performed on a Malvern Zetasizer Nano S equipped with a He-Ne (633 nm, 4 mW) laser and an Avalanche photodiode detector at an angle of 173 °. All DLS data were processed using Dispersion Technology Software (Malvern Instruments). Ultrasonication for the synthesis of the nanoparticles was performed on a VWR Ultrasonic Cleaner Model 75D.

Scanning electron microscopy and cryogenic scanning electron microscopy (SEM) was performed on a JEOL 2300 microscope operated at an acceleration voltage of 3 kV. 15 μl of polymersome solution was air-dried on a silicon wafer and placed on a conductive tape and attached to an aluminium sample block. The sample was then sputtered with Au/Pd alloy with a thickness of ~ 10 nm before visualization in the instrument.

Transmission electron microscopy (TEM) was performed on a JEOL 1010 microscope equipped with a CCD camera at an acceleration voltage of 60 kV. Sample specimens were prepared by placing a drop of the solution on a carbon-coated Cu grid (200 mesh, EM science) and air-dried. Processing and analysis of TEM images was performed with ImageJ, a program developed by the NIH and available as public domain software at http://rsbweb.nih.gov/ij/.

Cryogenic transmission microscopy The cryogenic transmission electron microscopy experiments were performed on a JEOL TEM 2100 microscope (JEOL, Japan).

High Magnetic Field Magnet The magnetic deformations and alignment of the supramolecular structures were performed at room temperature on a Duplex Bitter 20 T magnet with a size of the bore of 32 mm, operating under 5.80 MW power and with a field homogeneity of $1x10^{-3}$ in 1 cm DSV. The measurements were performed in the center of the field at 195 mm distance from ss top cover.

AF4-UV-Quels The asymmetric field flow fractionation – UV – Quels (AF4-UV-Quels) experiments were performed on a Wyatt Eclipse AF4 instrument connected to Shimadzu LC-20A Prominence system with Shimadzu CTO20A injector. The AF4 was further connected to the following detectors: a Shimadzu SPD20A UV detector, a Wyatt DAWN HELEOS II light scattering detector (MALS) using laser operating at 664.5 nm, a Wyatt Optilab Rex refractive index detector and a Quels detector installed at angle of 140.1^{o}. The AF4 channel was pre-washed with running solution of 5mM $NaNO_3$, which is also used for the separation. The hydrodynamic radius calculations was performed using Astra 6.1.1.The separation of the stomatocytes from the platinum nano-particles was performed on a AF4 short channel with regenerated cellulose (RC) 10 kDa membrane (Millipore) and spacer of 350 µm.

References

1 Kemp, J. C. *Polarized Light and its Interaction with Modulating Devices*. (HINDS International, Hillsboro, 1987).

2 Yu, Y. & Eisenberg, A. Control of Morphology through Polymer−Solvent Interactions in Crew-Cut Aggregates of Amphiphilic Block Copolymers. *J. Am. Chem. Soc.* **119**, 8383-8384 (1997).

3 Wilson, D. A., Nolte, R. J. M. & van Hest, J. C. M. Autonomous movement of platinum-loaded stomatocytes. *Nat. Chem.* **4**, 268-274 (2012).

4 Gupta, R. R. *Diamagnetische Suszeptibilität*. (Springer, Berlin, 1986).

5 Maret, G. & Dransfeld, K. *Strong and ultrastrong magnetic fields and their applications* Ch 4 (Springer-Verlag, Berlin, 1985).

6 Jasse, B. & Koenig, J. L. Fourier transform infrared study of uniaxially oriented atactic polystyrene. *J. Pol. Sci.* **17**, 799-810 (1979).

7 Vancso, G., Snétivy, D. & Tomka, I. Structural changes during polystyrene orientation: A study of optical birefringence and wide angle X-ray scattering. *J. Appl. Pol. Sci.* **42**, 1351-1359 (1991).

8 Lagowski, J. B., Csizmadia, I. G. & Vancso, G. J. Polystyrene models. II. Ab initio study of isobutylbenzene. *Int. J. Quant. Chem.* **43**, 595-623 (1992).

9. Giddings, J. C., Field-Flow Fractionation: Analysis of Macromolecular, Colloidal and Particulate Materials. *Science* **260**, 1456-1465 (1993)

10. Gigault J., Pettibone, J. M., Schmitt, C.,& Hackley, V. A., Rational strategy for characterization of nanoscale particles by asymmetric-flow field flow fractionation: A tutorial. *Anal. Chim. Acta* **809**, 9-24 (2014)
